# Understanding polymer encapsulation of enzymes: a dissipative particle dynamics simulation study on the regulation of structural characteristics of polymer nanocapsules†

**DOI:** 10.1039/d5sc02655e

**Published:** 2025-07-23

**Authors:** Bin Li, Bin Xu, Huimin Gao, Zhong-Yuan Lu

**Affiliations:** a State Key Laboratory of Supramolecular Structure and Materials, College of Chemistry, Jilin University China xubin@jlu.edu.cn gaohuimin@jlu.edu.cn luzhy@jlu.edu.cn

## Abstract

Enzymes play a crucial role as catalysts in biological processes, and enzyme therapy—utilizing biological enzymes—has gained significant attention for disease treatment. However, a critical challenge in enzyme therapy is the effective delivery of exogenous enzymes while maintaining their catalytic activity. Encapsulating enzymes in polymers offers a promising strategy to enhance their stability, prolong their half-life in the bloodstream, and improve biocompatibility. In this study, we employ dissipative particle dynamics (DPD) simulations combined with a reaction model to investigate the polymerization dynamics and the formation of a polymer nanocapsule around a nanoparticle that models an enzyme under mild reaction conditions. Our results show that the formation of a well-structured polymer nanocapsule depends on the strong attraction between monomers and the nanoparticle surface, low hydrophobicity, moderate polymerization rates, and weak chain stiffness. To optimize polymer nanocapsule preparation, we also examine the ratio of initiator to crosslinker at different monomer concentrations, identifying conditions that lead to a well-constructed polymer nanocapsule with high monomer participation. Our model is adaptable to various enzyme and monomer types by modifying their structures and properties, offering valuable insights for the future design of polymer nanocapsules in enzyme delivery.

## Abstract 2

Monomer adsorption capability, polymerization probability, chain rigidity, and reactant concentration synergistically regulate the formation of enzyme-polymer nanocapsules.

## Graphical abstract

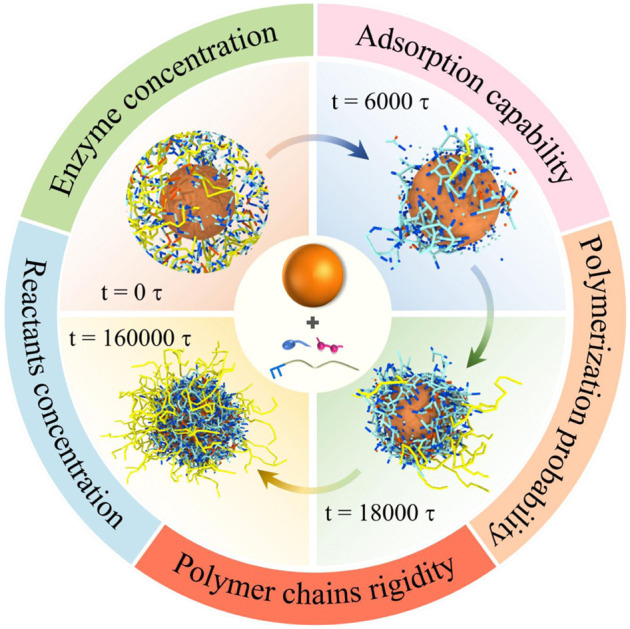

## 1 Introduction

Proteins are fundamental components of life, playing essential roles in maintaining cellular structure, catalyzing biochemical reactions, transmitting information, transporting substances, and defending against diseases.^1–3^ As functional biomolecules, enzymes act as highly specific catalysts in complex cellular processes, performing key biological functions.^4–6^ Recently, enzyme therapy has gained considerable attention for its potential to treat a range of disorders, including cancer, tumors, autoimmune diseases, and metabolic disorders.^7–11^ This therapeutic approach is favored for its high specificity, well-defined mechanisms of action, excellent biocompatibility, high catalytic efficiency, and minimal side effects.^12–15^ However, the clinical application of enzyme therapy is still hindered by challenges such as enzyme instability, low cellular permeability, and high immunogenicity.^11,13,16,17^

To overcome these limitations, various strategies have been developed to improve enzyme delivery. These include utilizing inorganic materials^18,19^ (such as carbon nanotubes, quantum dots, and nanoparticles), employing proteins as delivery carriers,^20,21^ and encapsulating enzymes within liposomes or polymers.^13,22,23^ Inorganic nanoparticles can deliver enzymes both on their surfaces and within their structures, offering enhanced structural stability. However, they tend to be inflexible and less biocompatible.^24^ Protein-based delivery carriers, while potentially more biocompatible, often face issues such as degradation by proteases in the body, complicating enzyme delivery. A promising alternative is to encapsulate enzymes within polymeric shells, as this approach not only protects the enzyme's folded state but also facilitates further functionalization of the polymer, enabling a range of multifunctional applications.^12,25^

Enzyme-polymer nanocapsules (polymer nanocapsules) represent a promising class of delivery systems. These nanocapsules are typically prepared through physical adsorption or covalent binding.^12,26^ Physical adsorption relies on non-covalent interactions, such as ion–ion interactions, hydrogen bonding, van der Waals forces, and hydrophobic interactions, to bind the polymer to the enzyme surface. For instance, Lv *et al.*^13^ developed guanidinium-rich polymer analogs, which interact strongly with the negatively charged carboxylate residues on enzymes through salt-bridging.^27^ Their research demonstrated that guanidino-π interactions in systems containing aromatic rings stabilize polymer/enzyme complexes, leading to effective cytoplasmic protein delivery. Compared to physical adsorption, covalent binding offers greater structural stability in the formation of enzyme-polymer complexes. For example, Wang *et al.* successfully encapsulated nerve growth factor within a polymer shell using acrylamide monomers and degradable crosslinkers, addressing concerns related to enzyme stability and clinical applicability.^28^ Similarly, hydrogen peroxide nanocapsules have been synthesized through *in situ* free radical polymerization, effectively mitigating the production of reactive oxygen species during viral infections and thereby protecting tissues from oxidative damage.^23^ However, covalent coupling methods often require genetic engineering or chemical modification of the proteins being loaded, which can involve complex synthesis and purification processes that may alter protein functionality.^29–31^

Additionally, Xu *et al.* developed carbon dot nanocapsules by utilizing electrostatic interactions between 2-methacryloyloxyethyl phosphorylcholine (MPC), *N*,*N*′-methylene bisacrylamide (BIS), and carbon dots.^32^ Their approach, which combined monomer enrichment followed by free radical polymerization, successfully integrated the advantages of both physical adsorption and chemical coupling. This method significantly enhances the retention time of the nanocapsule in the body. To better preserve enzyme activity and avoid altering its native structure, it would be ideal to design polymerizable monomers that can adsorb onto the enzyme surface without modifying the enzyme itself. These monomers will then undergo polymerization to form a protective polymer shell around the enzyme, enhancing its stability.

Inorganic nanoparticles and globular proteins exhibit significant differences in their internal structures. However, they share similarities in overall size, surface charge, and shape. Nanoparticles can effectively mimic proteins through the incorporation of functional groups on their surfaces.^33^ With their enhanced stability and versatile design, protein-mimicking nanoparticles demonstrate considerable potential for a wide range of applications.^34,35^ For example, luo *et al.* used gold-based nanomaterials to reproduce peroxidase-like catalytic activity.^36^ Shu *et al.* employed the means of amino-functionalized silica nanoparticles to anchor template proteins on the surface of the nanoparticles, and used physical adsorption to encapsulate the nanoparticles in layers of polysaccharide chains, thus preparation to obtain the nanocapsules.^37^

**Scheme 1:**
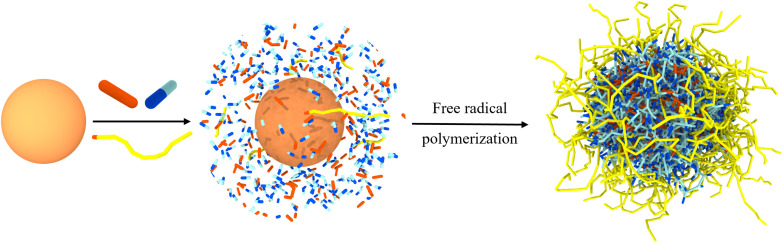
Synthesis of *n*(sphere). The *n*(sphere) is synthesized through free radical polymerization of monomers around a spherical particle in this study. In the schematic, the enzyme is represented as a simple spherical particle (O/N) colored in golden yellow. Blue (dark and light) and pink represent the reactive monomers (A-B) and crosslinkers (C-C), respectively. The light-yellow chain segments represent hydrophilic chains of length 10 (*E*_10_), which are modified with a polymerizable bead (C) at the terminal group. Solvent (W) and initiators (I) are omitted for clarity.

In this study, we omit the complex details of a specific enzyme, such as its surface charge or amino acid distribution. As illustrated in Scheme 1, we represent the enzyme as a spherical particle (O/N, golden yellow), as most enzymes adopt a globular protein structure. The diameter of globular protein molecules usually ranges from a few nanometers to several tens of nanometers.^38–40^ Based on this, we chose a moderate value (*r* = 5.0 *r*_c_, the radius of the sphere, with *r*_c_ ≅ 1.0 nm) as our representing size parameter for globule proteins. Monomers, crosslinkers and initiators are reactive monomers, crosslinking agents, initiators, *etc.* are indicated correspondingly. Additionally, we incorporate hyprohilic chains (*i.e.*, polyethylene glycol (PEG)) modifications into some of the polymeric monomers to improve the dispersibility of the nanocapsules and reduce the risk of gel formation.^41^ This modification not only enhances the preparation of polymer nanocapsule at higher concentrations but also makes the approach more feasible for practical applications.^42,43^ We employ dissipative particle dynamics (DPD) simulations combined with a reaction model to systematically investigate the properties of these monomers and the encapsulation process of enzyme-polymer nanocapsules under mild conditions (see Model and Simulation details in the ESI†). Through this analysis, we optimize the conditions for preparing well-structured polymer nanocapsules. We believe that these findings will provide valuable insights into the experimental preparation of enzyme-polymer nanocapsules and contribute to their potential clinical use.

## 2 Results and discussion

### 2.1 Effect of monomer properties on the structure of *n*(sphere)

In our simulations, following free radical polymerization, a thin, protective polymer shell forms on the surface of the sphere, resulting in the polymer nanocapsule (Scheme 1), which will be referred to as *n*(sphere) hereafter. Firstly, we systematically investigated the influence of monomer properties on the structural formation of *n*(sphere) through a series of controlled simulations. We performed extensive trial simulations with varying feeding ratios to optimize the reactant combination. Based on these simulations, we established an optimal stoichiometric ratio of *n*_(M)_ : *n*_(C)_ : *n*_(I)_ = 75 : 14 : 1 for the key reactive components, corresponding to a total of 1500 monomers within the simulation box. The system was further modified through the incorporation of hydrophilic chains functionalized with polymerizable beads, specifically employing CE_10_ as the structural unit at a concentration of 0.00519 mol L^−1^ (see Fig. S1† for detailed structural information).

**Fig. 1:**
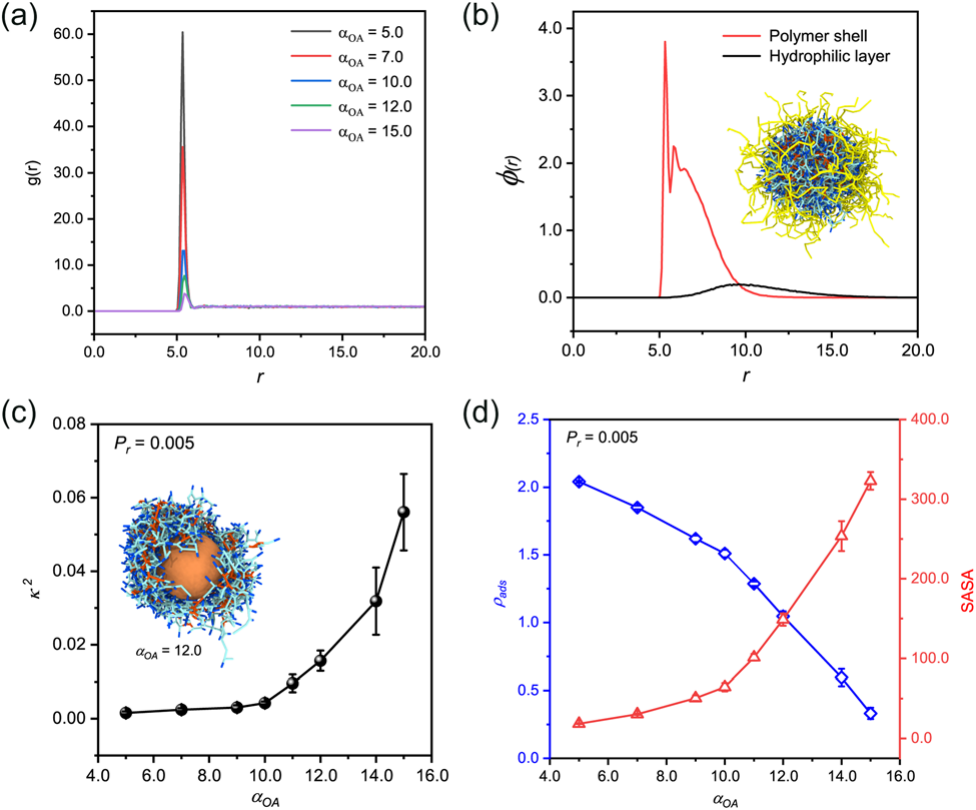
Structural characterization and adsorption-dependent properties of stabilized *n*(sphere) assemblies. (a) Radial distribution function (RDF) profiles of monomer (A-B) at varying adsorption capacities (*α*_OA_) in the pre-polymerization state. (b) A radial density profile of well-defined n(sphere) structures under optimal conditions (*α*_OA_ = 6.0, *P*_r_ = 0.005), with the corresponding conformations inserted. (c) Correlation between relative shape anisotropy (*κ*^2^) and adsorption interaction strength (*α*_OA_) at constant reaction probability (*P*_r_ = 0.005). The inbuilt image corresponds to the asymmetric *n*(sphere) conformation at *α*_OA_ = 12.0. The outer hydrophilic chain (*E*_10_) has been omitted for clarity of the image. (d) Dual-parameter analysis showing the interdependence of solvent accessible surface area (SASA) and monomer adsorption density (*ρ*_ads_) as functions of *α*_OA_. All systems were maintained at a fixed stoichiometric ratio of *n*_(M)_ : *n*_(C)_ : *n*_(I)_ = 75 : 14 : 1 (monomer: crosslinker: initiator). Visual representations employ the following color: central sphere (O, gold), crosslinkers (C-C, pink), monomer adsorbable beads (A, dark blue), polymerizable beads (B, light blue), and hydrophilic chains (*E*_10_, yellow). Solvent molecules are omitted from visualization for clarity.

After equilibrium, we quantitatively analyzed the monomer distribution relative to the spherical nanoparticle under varying adsorption strengths of bead A (Fig. 1(a)). The radial distribution function (RDF) analysis revealed that monomers preferentially accumulated at the nanoparticle surface. This adsorption phenomenon exhibited a positive correlation with the enhanced adsorption capacity of bead A, while maintaining a homogeneous distribution in regions distal to the spherical nanoparticle.

Upon initiating the polymerization process *via* the reaction model, we successfully obtained the *n*(sphere) structure. Density distribution analysis of the *n*(sphere) structure (Fig. 1(b)) demonstrated a characteristic spatial arrangement: as the radial distance from the spherical nanoparticle increased, we could observe sequential density peaks corresponding to the polymer chains followed by the outermost hydrophilic chains. The embedded image shows a typical *n*(sphere) structure after removal of unreacted species from the system. This layered structure provides valuable insights into the spatial distribution of different components in the *n*(sphere) system.

#### 2.1.1 Increased monomer adsorption capacity promotes the formation of well-ordered and stable *n*(sphere)

The designed monomer in our study consists of two functional components: an adsorbable bead (A) and a polymerizable bead (B). We maintained the intrinsic properties of polymerizable bead B, particularly its reaction probability, while systematically modulating the adsorption strength of bead A through adjustments of the interaction parameter (*α*_OA_) between bead A and the central sphere O. This parameter optimization allowed us to investigate its influence on the relative shape anisotropy (*κ*^2^) of the *n*(sphere) nanostructures.

Through systematic simulations with a fixed reaction probability (*P*_r_) of 0.005,^44,45^ we observed a positive correlation between increasing *α*_OA_ and *κ*^2^ of the *n*(sphere) structures (Fig. 1(c)). This relationship indicates that reduced adsorption capacity of bead A impedes the formation of well-defined nanocapsules. Our results also demonstrate that fully encapsulated nanocapsules with favorable morphological characteristics can still be achieved, as evidenced by *κ*^2^ values approaching zero (Fig. S2†).

Notably, when *α*_OA_ exceeds a threshold of 10.0, we observed a morphological transition where the polymer shell becomes asymmetrically distributed, preferentially accumulating on one side of the sphere (insert image in Fig. 1(c) and S2†). This structural rearrangement results in partial exposure of the central sphere to the solvent environment, which would compromise the protective function for real protein applications. Based on these findings, we established an optimal operational range for the adsorption capability of bead A at 3.0 ≤ *α*_OA_ < 10.0 (−6.73 < *χ*_OA_ ≤ −4.59). This non-bond interaction is validated in DPD simulations of hydroxyl-containing and amide-containing systems.^46^ The values indicate the presence of strong affinity between the beads by hydrogen bonding or electrostatic attraction.^47^ Furthermore, we identified the relative shape anisotropy value corresponding to *α*_OA_ = 10.0 (*κ*^2^ = 0.004) as the critical threshold for determining the feasibility of preparing regularized nanocapsule structures.

We also conducted a comprehensive characterization of the solvent accessible surface area (SASA) and adsorption density (*ρ*_ads_) across various conformational states of the nanosphere (Fig. 1(d)). The analysis reveals an inverse relationship between *ρ*_ads_ and *α*_OA_, demonstrating that the adsorption density systematically decreases with increasing *α*_OA_. Through measurements using a probe with a standardized radius of 0.1 nm, we found a positive correlation between SASA values and *α*_OA_ parameters. These correlations provide mechanistic insight: enhanced monomer adsorption capacity facilitates the formation of denser polymer networks on the sphere surface, which subsequently creates a more effective barrier against probe penetration. The resulting reduction in SASA values has direct implications for catalytic applications, as it influences the accessibility of catalytic substrates to the active sites of real proteins.

These findings underscore the importance of a dual-parameter optimization strategy when selecting adsorbable beads for monomer design. Specifically, the selection must simultaneously consider the bead capacity to achieve complete encapsulation and its influence on substrate accessibility. This balance is needed to ensure optimal catalytic performance while maintaining structural integrity.

#### 2.1.2 Moderate reaction probability balances polymer shell growth and structural regularity of *n*(sphere)

**Fig. 2:**
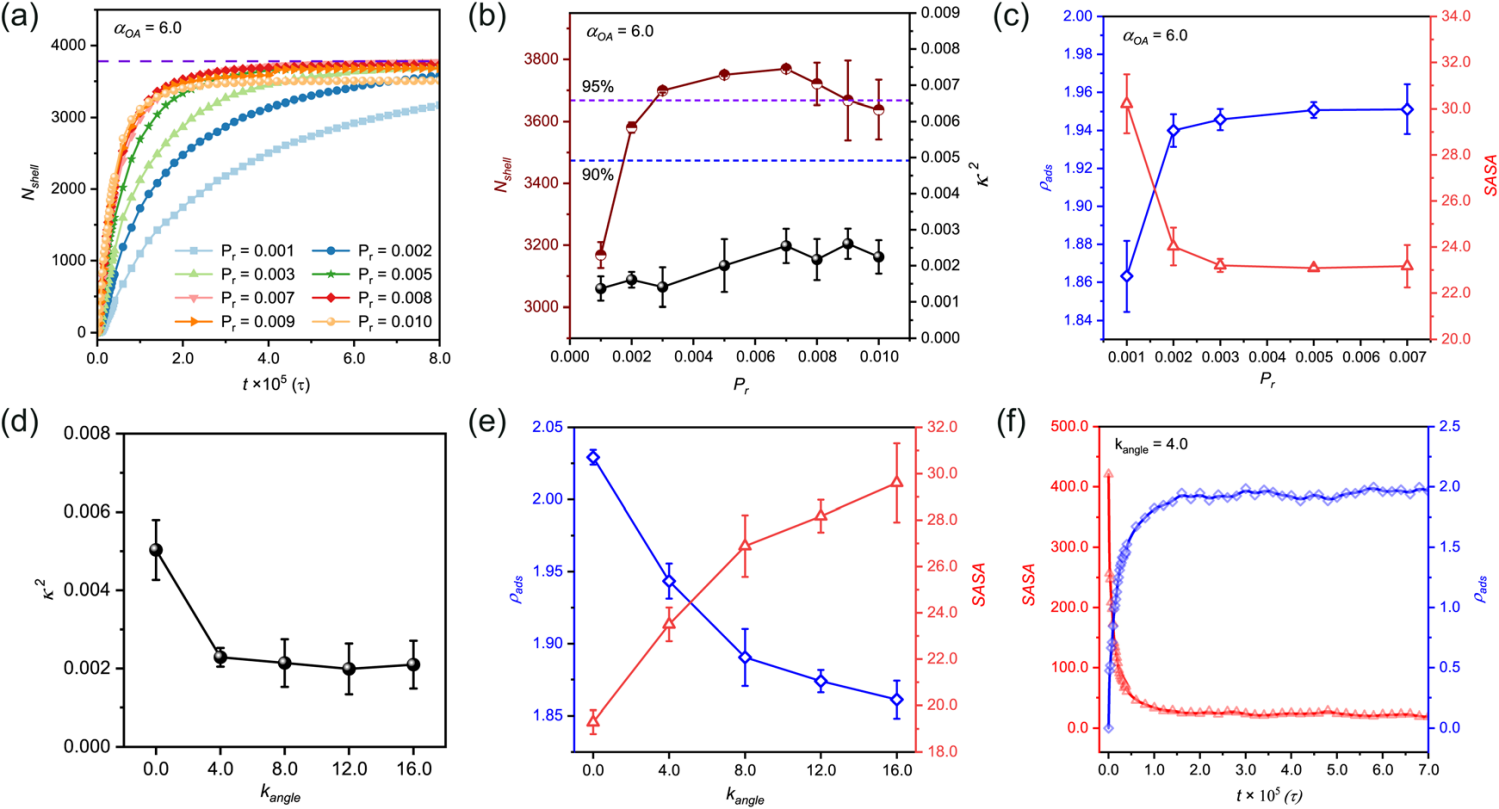
Influence of reaction probability and chain rigidity on structural parameters and morphological characteristics. (a) Temporal evolution of the number of beads in polymer shell (*N*_shell_) at varying reaction probabilities (*P*_r_) with fixed adsorption strength (*α*_OA_ = 6.0). (b) Correlation between stabilized *N*_shell_ values and corresponding relative shape anisotropy (*κ*^2^) across different *P*_r_ values at *α*_OA_ = 6.0, with dashed lines indicating distinct monomer utilization efficiency. (c) Dual-parameter analysis of SASA and *κ*^2^ as functions of reaction probability at *α*_OA_ = 6.0. (d) Dependence of *κ*^2^ on chain rigidity (*k*_angle_) under optimized conditions (*α*_OA_ = 6.0, *P*_r_ = 0.005). (e) Quantitative assessment of adsorption density (*ρ*_ads_) and SASA with various chain rigidities. (f) Time evolution of SASA and *ρ*_ads_ at an intermediate chain rigidity (*k*_angle_ = 4.0). The molar ratios of monomers, crosslinkers, and initiators in panels (a) to (f) are 75 : 14 : 1, namely *n*_(M)_ : *n*_(C)_ : *n*_(I)_ = 75 : 14 : 1.

In our study, we introduce the reaction probability (*P*_r_) to characterize the reaction rate of the system. Through investigation of the number of beads in the polymer shell (*N*_shell_) as a function of polymerization time at varying *P*_r_ values (while maintaining constant adsorption capacity at *α*_OA_ = 6.0, Fig. 2(a)), we presented growth kinetics of *n*(sphere). The theoretical maximum *N*_shell_, represented by the purple dashed line, corresponds to complete reactant participation.

Our simulation data reveal that in the early polymerization stage, the growth rate of the polymer shell increases with increasing *P*_r_. Simultaneously monitoring *N*_shell_ and *κ*^2^ (Fig. 2(b)) provides quantitative insights into structural evolution of *n*(sphere). At *P*_r_ = 0.001, the system demonstrates insufficient reactivity (Fig. S3†), resulting in low monomer conversion efficiency. As *P*_r_ increases to 0.002, bead participation improves slightly but does not reach the optimal. Notably, when *P*_r_ exceeds 0.007, the system shows diverse configurations, with some structures stabilizing as solvent-dispersed micelles rather than contributing to shell formation (Fig. S3 and S4†). This phenomenon arises from kinetic competition between chain propagation and diffusion: rapid polymerization at high *P*_r_ values promotes localized chain growth near randomly distributed initiators, while distal chains undergo independent micellization before reaching the central sphere.

Therefore, we identified the optimal reaction probability window as 0.003 ≤ *P*_r_ ≤ 0.007. Within this range, the system achieves both high monomer utilization efficiency (Fig. 2(b)) and well-ordered nanocapsule formation (Fig. S3†). These findings emphasize the importance of maintaining moderate reaction probabilities to ensure optimal structural integrity and morphological control in nanocapsule synthesis.

We further investigated the dual-parameter variations of *ρ*_ads_ and SASA as functions of *P*_r_ (Fig. 2(c)). The analysis revealed that polymer shells with lower *P*_r_ values exhibited greater structural porosity, corresponding to diminished adsorption densities. As the reaction rate increased, the shell structure underwent progressive compaction, driving a corresponding rise in adsorption density that eventually reached a plateau. This tighter structure also made it harder for probe molecules to penetrate, which reduced the SASA values and kept them stable at higher *P*_r_ levels.

In a practical system, the reaction probability (*P*_r_) can be expressed as an Arrhenius-type equation, *P*_r_ = *A* exp(−*E*_a_/(*k*_B_*T*)), where *A* is a modifying factor, *E*_a_ is the activation energy of the reaction, *k*_B_ is the Boltzmann constant, and *T* is the absolute temperature. Lu *et al.* used a general free radical polymerization activation energy value to estimate the modifying factor *A*, which was calculated to be *A* = 2.2 × 10^5^.^48^ We used this value to estimate the activation energy of our system. When *P*_r_ = 0.005, the calculated *E*_a_ was about 43 kJ mol^−1^. For experimental systems, this value is mostly common for the activation energy for the polymerization of vinyl-based monomers.^49,50^

#### 2.1.3 Optimal chain rigidity regulates structural uniformity and functional stability of *n*(sphere)

It has been established that the stiffness of polymer chains influences the size of nanocapsules and plays a critical role in regulating their cellular uptake during circulation in the bloodstream.^51,52^ Based on this, we investigated how the properties of polymer chains formed after polymerization influence the configuration of *n*(sphere). Our simulations were conducted under optimized conditions (*α*_OA_ = 6.0, *P*_r_ = 0.005), while systematically modulating the angle rigidity factor (*k*_angle_) from 0.0 (fully flexible chains) to 16.0 (semi-rigid chains)^44,53^ to assess the impact of polymer chain rigidity on *n*(sphere) structure.

The results indicate that the *κ*^2^ value demonstrates a trend of initially decreasing, and reaching a steady state (Fig. 2(d)). At *k*_angle_ = 0, the observed *κ*^2^ values (>0.004) surpassed our established regularity threshold, indicating suboptimal structural organization. In this fully flexible chain state, the polymer chains exhibit excessive degrees of freedom, leading to strong conformational fluctuations. This molecular-level disorder manifests as inefficient crosslinking site binding and the formation of irregular nanocapsules with undesirable cluster branching (Fig. S5†).

The introduction of chain rigidity induces a structural transition by largely constraining chain mobility. From a thermodynamic perspective, this rigidity enhancement promotes system stabilization through decreasing free energy. The semi-rigid chains demonstrate improved self-assembly capabilities, adopting lower energy conformations that facilitate the formation of more regular nanocapsule structures. Excessive chain stiffness may lead to over-compact conformations and local energy traps, ultimately impeding *n*(sphere) structural optimization. These findings establish that while chain flexibility is essential for initial molecular reorganization, moderate rigidity is crucial for achieving well-ordered nanocapsule architectures.

To further characterize the structural evolution, we conducted systematic measurements of *ρ*_ads_ and SASA using a standardized probe size of 0.1 nm for stabilized configurations (Fig. 2(e)). Quantitative analysis revealed a distinct inverse correlation between chain rigidity and adsorption density, with *ρ*_ads_ values showing a progressive decrease as *k*_angle_ increased. Conversely, SASA values exhibited a consistent positive correlation with chain rigidity, demonstrating an opposite trend. This behavior can be mechanistically explained by considering the molecular-level packing dynamics in the flexible chain regime (low *k*_angle_ values). The enhanced conformational adaptability of flexible chains enables greater monomer accommodation on the nanosphere surface, while simultaneously facilitating denser surface packing. This dual effect significantly reduces the accessible surface area for probes, leading to diminished SASA values in the flexible chain regime.

To gain deeper insights into the temporal evolution of these parameters, we performed time-resolved monitoring at an intermediate rigidity (*k*_angle_ = 4.0, Fig. 2(f)). It shows that *ρ*_ads_ values undergo rapid increase followed by leveling off, reflecting the progressive saturation of adsorption sites. Simultaneously, SASA values exhibit a gradual decline as the polymerization progresses, eventually reaching equilibrium as the nanosphere surface becomes fully occupied by reacted monomers. These complementary kinetic profiles indicate the importance of dynamic balance between surface adsorption and accessibility in nanocapsule formation.

Our systematic investigation reveals that polymer chain flexibility serves as a crucial role in governing the structural characteristics of the *n*(sphere). Through controlled modulation of chain rigidity, we identified that an optimal degree of stiffness enhancement not only improves shell regularity but also achieves a balanced optimization of *ρ*_ads_ and SASA. The established correlations between chain rigidity and nanocapsule morphology provide essential design principles for engineering advanced nanocapsule-based drug delivery systems with tailored structural and functional properties.

Additionally, we can judge the flexibility of the polymer chains by using the persistence length as a bridge between the simulation and the experimental system. The so-called persistence length (*l*_p_), which is one of the microscopic parameters characterizing the intrachain length scales and the chain stiffness, can be derived from orientation correlation function: 〈cos *θ*(*s*)〉 = exp(−*sl*_b_/*l*_p_).^54^ In our simulation, we calculated *l*_p_ to be approximately 1.56 nm (see page 4 of the ESI† for specific details), which falls into the flexible chain interval.^55–57^

#### 2.1.4 Dual parameter optimization of adsorption-hydrophobicity equilibrium enables precise design of *n*(shpere)

As fundamental non-covalent driving forces, hydrophobic interactions critically govern the supramolecular assembly of polymer chains in solution. Through systematic modulation of hydrophobic moieties on bead B, (*i.e.*, by manipulating repulsive parameter *α*_WB_), we regulated hydrophobic interactions and ultimately tuned the morphological outcome of assembled structures. This investigation builds upon our previous findings demonstrating the influence of monomer adsorption capacity (*α*_OA_) on determining the polymer shell architecture.

**Fig. 3:**
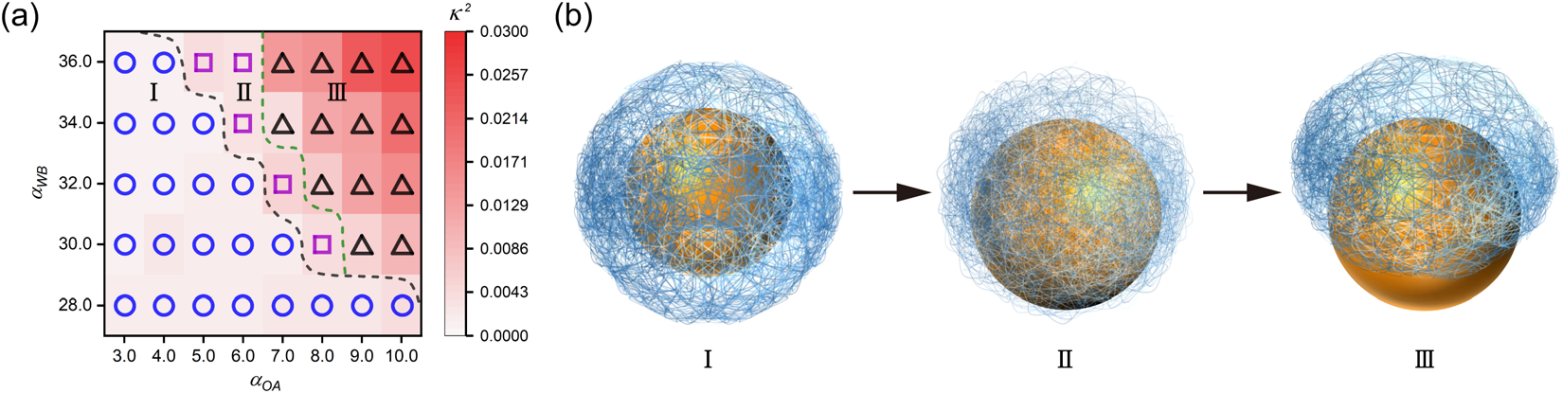
Morphological characteristics in *n*(sphere) assemblies. (a) Systematic variation of adsorption capacity (*α*_OA_: 3.0–10.0) and hydrophobicity (*α*_WB_: 28.0–36.0) modulates nanocapsule anisotropy (*κ*^2^), with outlined thresholds for morphological transitions. (b) Schematics for delineating three characteristic morphological regimes: (I) fully encapsulated structures, (II) distorted encapsulations, and (III) partially exposed structures. All systems were prepared at the same stoichiometry under optimized parameters (*P*_r_ = 0.005). Schematic representations emphasize core–shell architecture (gold: central nanosphere, light blue: polymer network) with peripheral chains omitted for clarity.

We established a phase diagram (Fig. 3) mapping the cooperative effects of adsorption capacity (*α*_OA_) and hydrophobicity (*α*_WB_) on nanocapsule conformational regularity. The parameter space is categorized into three distinct regimes corresponding to characteristic structural morphologies. For visual clarity, schematic representations as shown in Fig. 3(b) exclude peripheral hydrophilic chains, depicting the central nanosphere in gold and polymer shell networks in light blue.

Simulation snapshots (Fig. S6†) reveal a progressive structural transition: decreasing bead A adsorption capacity coupled with increasing bead B hydrophobicity induces gradual solvent exposure of the central nanosphere. This can be attributed to the fact that, hydrophobic interactions dominate in a system characterized by weak adsorption strength and strong hydrophobicity, prompting the polymer chains to congregate in specific regions to minimize contact with the solvent, ultimately resulting in a polymer shell structure with asymmetric distribution. In the work of chen *et al.*,^58^ a similar finding was made by modulating the relative concentration of hydrophilic and hydrophobic ligands, a shift in the core/shell structure from concentric to slightly eccentric to highly eccentric during encapsulation of Au nanoparticles is observed.

These findings demonstrate morphological control through dual parameter optimization: (1) enhanced bead A adsorption capacity promotes surface-localized assembly, while (2) reduced bead B hydrophobicity minimizes intramolecular aggregation. This optimization enables the formation of well-defined nanocapsules, providing guidelines for engineering functional polymer assemblies with tailored solvent interactions.

#### 2.1.5 Comparison of monomer properties in practical applications

To design a functional monomer for nanocapsule formation, we considered two distinct components in one monomer, *i.e.*, in the model, an adsorbable bead A and a polymerizable bead B, which serve as the anchoring unit and the polymerization unit, respectively. For the successful formation of a polymer shell on a specific protein surface, effective anchoring of bead A is essential. Therefore, we first examined the chemical composition of the adsorbable bead (A) independently. In this study, bovine serum albumin (BSA) was used as a model protein to systematically investigate the interaction patterns between protein surfaces and adsorbable bead (A) through all-atom molecular dynamics (AAMD) simulations. The results indicate that monomers based on acetamide derivatives exhibit strong and stable adsorption affinity towards polar and charged functional groups (such as carboxyl, amino, and hydroxyl groups) present on the BSA surface (Fig. S19† and the detailed calculation method in ESI†). Bifunctional, strongly polar groups (*e.g.*, NH_2_COCH_2_COOH) that enable multiple hydrogen bonds and salt bridges show superior adsorption onto protein surfaces. Thus, selecting monomers with multifunctional polar modifications is optimal for stable anchoring. For the design of the polymerization bead (B), we determined that the polymer chain stiffness should lie within a semi-flexible regime (persistence length approximately equal to 0.3–2.0 nm) and the activation energy should be roughly equal to 43 KJ mol^−1^. Based on these criteria, simple vinyl-based monomers can be employed in practice. During experiments, fine-tuning the reaction temperature enables precise control of the polymerization rate and optimal formation of the nanocapsule shell.

### 2.2 Hierarchical assembly mechanism of *n*(sphere)'s polymer shell: surface-constrained polymerization mediated by competitive growth pathway

**Fig. 4:**
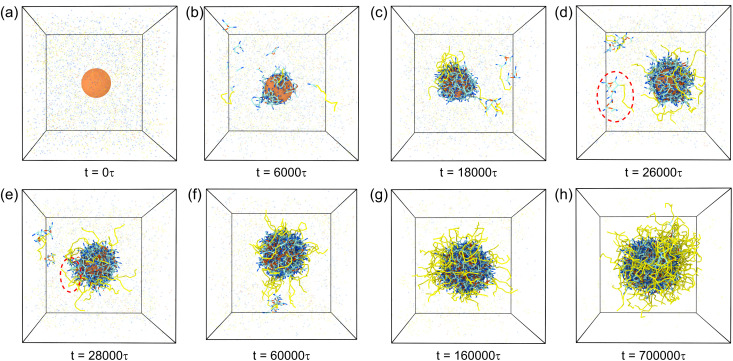
Time-resolved morphological evolution of *n*(sphere) assemblies during polymerization. Snapshots (a–h) illustrate the growth process under optimized stoichiometric conditions with fixed CE_10_ hydrophilic chain concentration (0.00519 mol L^−1^). They show the sequential stages of polymer shell formation, including initial nucleation, competitive growth *via* dual polymerization pathways, and stable shell maturation. Reaction simulation parameters were maintained at *α*_OA_ = 6.0 and *P*_r_ = 0.005 throughout the process. Temporal progression is indicated below each snapshot. For visual clarity, solvent beads are omitted. Color scheme is the same as in Fig. 1: central nanosphere (gold), crosslinkers (pink), adsorption beads (dark blue), polymerization beads (light blue), and hydrophilic chains (yellow).

The mechanistic understanding of polymerization dynamics at enzyme–mimetic interfaces represents a fundamental aspect of nanocapsule engineering. Through high-temporal-resolution simulation analysis, we have systematically mapped the growth kinetics of polymer shells in different stages. Our visualization strategy explicitly distinguishes reacted polymer segments from unreacted precursors, represented as discrete beads (Fig. 4).

The polymerization cascade initiates at *t* = 0 *τ*, corresponding to the initial state (Fig. 4(a)). With time increasing, selective monomer adsorption through specific A–O interactions (*α*_OA_ = 6.0) leads to the formation of surface-anchored oligomers (Fig. 4(b)). During this “nucleation” phase, distal reactants maintain homogeneous dispersion in the solvent. As the reaction progresses to *t* = 18 000 *τ*, we observe the emergence of dual polymerization pathways: (1) surface-confined chain propagation and (2) solvent-mediated oligomerization (Fig. 4(c)).

A structural transition occurs as solvent-born short chains undergo surface-directed migration, driven by A-O interactions (*α*_OA_), ultimately integrating into the growing shell (Fig. 4(d) and (e)). Notably, kinetic competition becomes apparent at elevated reaction probabilities, where accelerated solvent-phase polymerization induces premature chain stabilization through hydrophilic corona formation, effectively passivating reactive chain termini (Fig. S4†).

The system enters the maturation stage at *t* = 60 000 *τ*, marked by interchain reactions among solvent-dispersed oligomers (Fig. 4(f)). This stage evolves into chain-length-dependent growth dynamics by *t* = 160 000 *τ*, where extended polymer chains preferentially incorporate shell-embedded reactive sites, while residual monomers undergo continuous surface polymerization, resulting in progressive shell thickening (Fig. 4(g)). The resulting architecture precisely matches our design specifications, comprising a dense polymer matrix encapsulating the nanosphere core, and an outer hydrophilic stabilization layer. Finally, most reactants in the system participate in the reaction, ultimately achieving final nanocapsule conformation (Fig. 4(h)).

### 2.3 Effect of monomer concentration and stoichiometric ratio on *n*(sphere) conformation

Monomer concentration and stoichiometric ratio are vital for the morphology and efficiency of the synthesized *n*(sphere). Although higher monomer concentrations can accelerate reaction rates, conventional encapsulation strategies encounter challenges such as irreversible gelation at elevated enzyme concentrations and reduced monomer conversion efficiency, complicating enzyme functionalization. These challenges underscore the necessity for further research into optimizing polymerization through precise adjustments of monomer concentration and feeding ratio.

#### 2.3.1 Optimal initiator and crosslinker concentrations are essential to ensure efficient polymerization and the structural integrity of *n*(sphere)

**Fig. 5:**
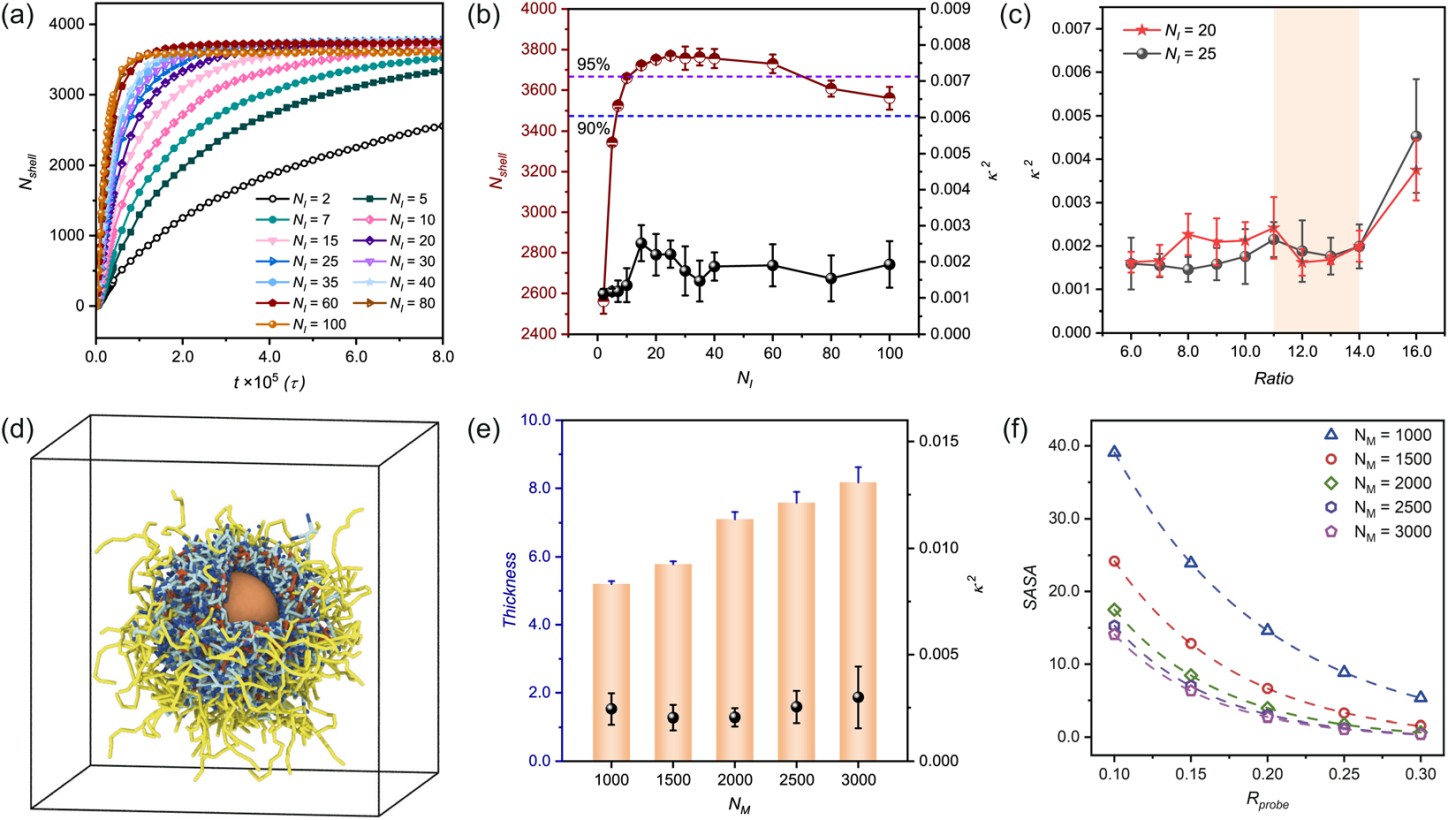
Composition-dependent morphological evolution of *n*(sphere) assemblies. (a) Temporal evolution of the number of beads in polymer shell (*N*_shell_) at varying initiator concentrations (*N*_I_) under fixed conditions (*n*_(C)_ : *n*_(I)_ = 14 : 1, *N*_M_ = 1500). (b) Dependence of relative shape anisotropy (*κ*^2^) and *N*_shell_ on different initiator concentrations. (c) Crosslinker concentration-dependent relative shape anisotropy(*κ*^2^) at optimal initiator concentrations (*N*_I_ = 20, 25), expressed *versus n*_(C)_ : *n*_(I)_ ratio. (d) Snapshot of *n*(sphere) morphology at *N*_M_ = 2000, revealing internal structural organization. (e) Monomer concentration effects on shell thickness and relative shape anisotropy (*κ*^2^). (f) Solvent accessibility (SASA) as a function of monomer concentration, with different probe sizes (symbols: simulation data; dashed line: fitted curve). In the simulation snapshot, solvent is not shown for clarity and the color settings are the same as those in Fig. 1. All simulations were conducted under standardized conditions (*α*_OA_ = 6.0, *P*_r_ = 0.005).

To establish optimal component stoichiometry, we maintained a fixed monomer concentration (in simulations *N*_M_ = 1500) under optimized reaction conditions (*α*_OA_ = 6.0, *P*_r_ = 0.005). Our systematic investigation first focused on initiator concentration effects on *n*(sphere) morphology. Temporal evolution of the number of beads in polymer shell (*N*_shell_) at varying initiator concentrations reveals distinct polymerization kinetics (Fig. 5(a)). Higher initiator concentrations accelerate polymerization through increased radicals, effectively enhancing monomer activation and chain propagation rates. This concentration-dependent behavior mirrors the effects observed in reaction probability modulation.

Through parallel simulations, we statistically evaluated stabilized conformations by quantifying both *N*_shell_ and *κ*^2^ across different initiator concentrations (Fig. 5(b)). While all conditions yielded low *κ*^2^ values (indicating high structural regularity), initiator concentration significantly influenced bead participation efficiency (*i.e.*, *N*_shell_). Insufficient initiator levels resulted in incomplete conversion within simulation timescales (Fig. S7a and b†), whereas excessive initiator content promoted competitive micellization in solvent, reducing shell incorporation efficiency (Fig. S7g and h†).

It is well known that effective free radical polymerization typically achieves a monomer conversion of 85–90% and can even be higher than 90%.^59,60^ Considering this, we selected two initiator concentrations (in practice *N*_I_ = 20, 25) within the optimal monomer conversion range (namely, above 95%) to investigate the effects of crosslinker concentration on the *n*(sphere) conformation (Fig. 5(c)).

Systematic variation of crosslinker concentration revealed an important balance: excessive crosslinker content (Ratio = *n*_(C)_ : *n*_(I)_ = 16.0) induced structural irregularities through asymmetric shell deformation, while insufficient crosslinking led to poor chain incorporation to form the polymer shell. At low crosslinker concentrations, although its effect on the regularity of *n*(sphere) is small (Fig. S8 and S9†). But in the curves characterising the thickness of the polymer shell, we can found: the shell thickness at low crosslinker concentrations is greater, indicating that the outer polymer chains are relatively more extended and flexible. As the concentration of crosslinker appropriate increases, a noticeable shrinkage in the shell thickness occurs, suggesting that a higher amount of crosslinkers enhances the crosslinking of the outer chains to the central shell (Fig. S10†). These findings establish an optimal crosslinker-to-initiator ratio range of 11 < *n*_(C)_ : *n*_(I)_ ≤ 14 for *N*_M_ = 1500, ensuring both structural integrity and efficient monomer utilization.

#### 2.3.2 Concentration-dependent engineering of *n*(sphere)'s shell under fixed stoichiometry conditions

Maintaining a constant feeding ratio, we systematically investigated the influence of monomer concentration on polymer shell characteristics. Fig. 5(d) presents a snapshot of *n*(sphere) morphology at *N*_M_ = 2000, revealing successful core encapsulation and internal structural organization within the polymer shell. Quantitative analysis of *κ*^2^ and shell thickness across varying monomer concentrations demonstrates consistency in nanocapsule morphology (Fig. 5(e)). Simulation snapshots (Fig. S11†) corroborate these findings, confirming minimal monomer concentration-dependent variations in structural regularity.

Complementary solvent accessibility studies (Fig. 5(f)) reveal dual dependence of SASA values: (1) for fixed monomer concentrations, SASA decreases with increasing probe size due to steric exclusion effects; (2) at constant probe dimensions, SASA reduction correlates with increasing monomer concentration, reflecting enhanced surface packing density. This inverse relationship between monomer concentration and solvent accessibility stems from denser polymer network formation, which effectively restricts probe penetration.

These findings establish a concentration-dependent design space for nanocapsule engineering, enabling optimization of shell thickness and substrate accessibility based on catalytic requirements. The demonstrated structural consistency across concentration variations suggests robust assembly characteristics, while the tunable solvent accessibility provides critical design parameters for catalytic applications.

#### 2.3.3 Concentration-adaptive stoichiometry optimization for extensible *n*(sphere) synthesis

While previous investigations employed fixed feeding ratios to examine reactant concentration effects, we recognize that optimal stoichiometry varies with concentration. To establish these relationships, we maintained a constant monomer-to-initiator ratio while systematically evaluating *κ*^2^ and reaction efficiency (*η*_react_) across different concentrations. When probing the initiator concentration on the structural regularity of the nanocapsules, we found that decreasing the initiator concentration has less effect on the *κ*^2^ of *n*(sphere) (Fig. 5(b)), but rather decreases the efficiency of the reaction preparation. To efficiently prepare polymer nanocapsules, we selected a relatively high initiator concentration (1.3 mol%) to complete the polymerization reaction (namely, *n*_(M)_ : *n*_(I)_ = 75 : 1). This initiator concentration is experimentally permissible and it has also been demonstrated that an appropriate increase in initiator concentration significantly reduces the polymerisation time without affecting the molar mass distribution of the polymer.^61^ All reported values represent averaged data from five independent simulation replicates.

**Fig. 6:**
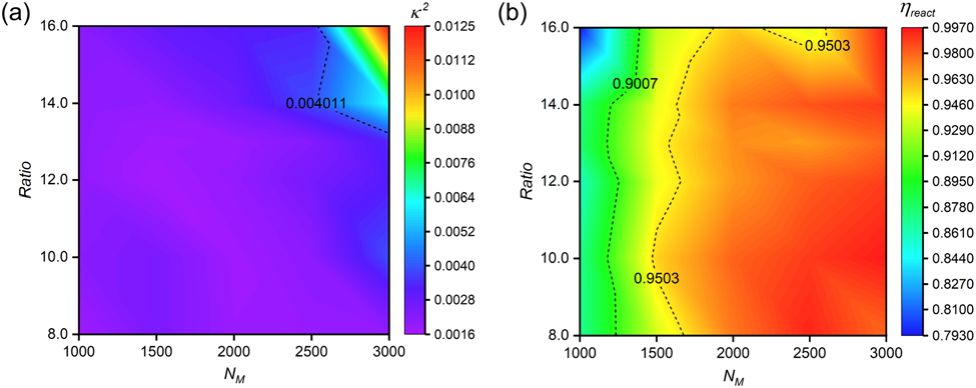
Concentration-dependent structural optimization and reaction efficiency. (a) Correlation between reactant concentrations and relative shape anisotropy (*κ*^2^) of *n*(sphere) assemblies. (b) Reaction efficiency (*η*_react_) as a function of monomer concentration at optimized stoichiometry. All systems maintain fixed *n*_(M)_ : *n*_(I)_ = 75 : 1, with crosslinker content expressed as *n*_(C)_ : *n*_(I)_ ratio. Simulations were conducted under standardized conditions (*α*_OA_ = 6.0, *P*_r_ = 0.005). Data points represent averaged values from five independent replicates.

Fig. 6(a) reveals a non-monotonic relationship between monomer and crosslinker concentrations, defining optimal concentration windows. Representative snapshots from the *N*_M_ = 3000 system (Fig. S12†) illustrate this concentration-dependent behavior: (1) at low crosslinker concentrations, polymer shells exhibit dispersed surface morphology; (2) optimal crosslinker content yields well-defined, regular structures; (3) excessive crosslinking induces structural asymmetry and reduced regularity. These findings emphasize the importance of precise stoichiometric control in nanocapsule synthesis.

Reaction efficiency analysis (Fig. 6(b)) demonstrates enhancement in bead participation (*η*_react_) with increasing monomer concentration at optimal crosslinker levels. This enhancement stems from increased reaction possibility among hydrophilic chains in monomer-rich environments. We establish *η*_react_ = 90% as the threshold for efficient nanocapsule formation with minimal material loss.

These results provide a comprehensive framework for concentration-dependent feeding ratio optimization, offering guidance for experimental synthesis of structurally regular nanocapsules. The established relationships between monomer concentration, crosslinker concentration, and reaction efficiency enable rational design of synthesis protocols tailored to specific concentration ranges.

Finally, in practical applications, achieving efficient large-scale production is often the primary objective. Therefore, exploring simulation studies on the preparation of nanocapsules in a high-concentration system is essential. We increased the number of nanospheres to investigate the conformational morphologies and distributions of *n*(sphere)s obtained at different concentrations. Maintaining identical reaction parameters (*α*_OA_ = 6.0, *P*_r_ = 0.005) established in single-sphere simulation studies. This approach enabled the successful preparation of both mono- and multi-encapsulated nanocapsules (Fig. S13†), demonstrating the system's scalability. Although we could not completely achieve well-distributed *n*(sphere) structures, we found that no inter-capsule crosslinking occurs in our systems. This significantly mitigates the risk of gel formation during high-concentration experimental preparations.

Quantitative analysis of encapsulation structures across concentration ranges (Fig. S14†) revealed two key trends: (1) nanocapsule polydispersity increases with nanosphere concentration, implying enhanced diversity in protein encapsulation structures; (2) despite increased proportion of larger nanocapsules at higher concentrations, single-encapsulated *n*(sphere) structures always dominate (>50%).

These findings highlight the practical relevance of our simulations, demonstrating that the established reaction parameters remain effective at elevated concentrations, and the system exhibits inherent resistance to gelation. This scalability analysis bridges the gap between fundamental studies and practical applications, offering valuable guidance for experimental optimization of high-concentration nanocapsule synthesis.

## 3 Conclusions

Through systematic dissipative particle dynamics (DPD) simulations, we have developed and characterized a novel class of functional monomers optimized for constructing protective polymer shells on nanosphere surface *via* free radical polymerization. Our findings establish suitable conditions for successful *n*(sphere) formation: strong monomer adsorption capacity, controlled hydrophobicity, moderate polymerization kinetics, and optimal chain rigidity. Comprehensive investigation of reaction stoichiometry reveals a well-defined window of feeding ratios that simultaneously maximize structural regularity and reaction efficiency, providing an economically viable pathway for nanocapsule synthesis.

The adaptability of our model enables precise modulation of polymer properties, offering opportunities for advanced optimization of enzyme-polymer nanocapsules. These fundamental insights not only advance our understanding of nanoscale encapsulation processes but also establish a platform for developing next-generation delivery systems. Future research directions should focus on: refining polymer architectures for specific biological applications, exploring dynamic encapsulation-release mechanisms, and translating these computational insights into experimental protocols for therapeutic enzyme delivery systems.

## Author contributions

B. X., H. G. and Z.-Y. L. directed the project. B. L. performed the simulations, analyzed the data, and wrote the manuscript. All authors participated in the discussion and preparation of the manuscript.

## Conflicts of interest

There are no conflicts to declare.

## Supplementary Material

SC-OLF-D5SC02655E-s001

SC-OLF-D5SC02655E-s002

## Data Availability

The data that support the fndings of this study are included in the main text and the ESI.†
