# Supplementary material for: Understanding polymer encapsulation of enzymes: a dissipative particle dynamics simulation study on the regulation of structural characteristics of polymer nanocapsules
Source: Chem Sci. 2025 Jul 23;16(34):15427–37. doi: 10.1039/d5sc02655e (PMC12305466; doi:10.1039/d5sc02655e)
Supplement: SC-016-D5SC02655E-s001 [file SC-016-D5SC02655E-s001.pdf]

Supplementary Information (SI) for Chemical Science.
This journal is © The Royal Society of Chemistry 2025

Electronic Supplementary Information

Understanding Polymer Encapsulation of Enzyme: A Dissipative Particle Dynamics Simulation Study on the Regulation of Structural Characteristics of Polymer Nanocapsule

Bin Li,[a] Bin Xu,*[a] Huimin Gao,*[a] and Zhong-Yuan Lu*[a]

[a] *State Key Laboratory of Supramolecular Structure and Materials, Institute of Theoretical Chemistry, Jilin University, Changchun 130023, China.*
E-mail: xubin@jlu.edu.cn; gaohuimin@jlu.edu.cn; luzhy@jlu.edu.cn

1 Model and Simulation Details

Dissipative particle dynamics (DPD) is a coarse-grained simulation method that can effectively incorporate the hydrodynamics of complex fluids while retaining the essential properties of the system's components[1,2]. In this study, we use the DPD simulation method[3–5] to explore the preparation conditions of polymer nanocapsule. In DPD, the time evolution of coarse-grained beads is governed by Newton's equations of motion[6],

$$\frac{d\boldsymbol{r}_i}{dt} = \boldsymbol{v}_i, \tag{S1}$$

and

$$m\frac{d\boldsymbol{v}_i}{dt} = \boldsymbol{f}_i, \tag{S2}$$

where $\boldsymbol{r}_i$, $\boldsymbol{v}_i$ and $\boldsymbol{f}_i$ are, respectively, the position, velocity and force on the i-th bead with mass m at time t. The force acting on bead i by bead j contains three non-bonded pairwise forces, i.e.,

$$f_i = \sum_{i \neq j} (\boldsymbol{F}^C_{ij} + \boldsymbol{F}^D_{ij} + \boldsymbol{F}^R_{ij}), \tag{S3}$$

including the conservative force $\boldsymbol{F}^C_{ij}$, dissipative force $\boldsymbol{F}^D_{ij}$, and random force $\boldsymbol{F}^R_{ij}$. They are given, respectively, as follows:

$$\boldsymbol{F}^C_{ij} = \begin{cases} \alpha_{ij}\left(1 - \frac{r_{ij}}{r_c}\right)\boldsymbol{e}_{ij} & r_{ij} < r_c, \\ 0 & r_{ij} \geq r_c, \end{cases} \tag{S4}$$

$$\boldsymbol{F}^D_{ij} = -\gamma\omega^D(r_{ij})(\boldsymbol{v}_{ij} \cdot \boldsymbol{e}_{ij})\boldsymbol{e}_{ij}, \tag{S5}$$

and

$$\boldsymbol{F}^R_{ij} = \sigma\omega^R(r_{ij})\xi_{ij}\Delta t^{-\frac{1}{2}}\boldsymbol{e}_{ij}, \tag{S6}$$

where $\boldsymbol{r}_{ij} = \boldsymbol{r}_i - \boldsymbol{r}_j$, $r_{ij} = |\boldsymbol{r}_{ij}|$, $\boldsymbol{e}_{ij} = \boldsymbol{r}_{ij}/r_{ij}$, $\boldsymbol{v}_{ij} = \boldsymbol{v}_i - \boldsymbol{v}_j$, α_{ij} represents the maximum repulsion between interacting beads i and j. ω^D and ω^R are weight functions for the dissipative and random forces, respectively. γ is a friction coefficient, which indicates the strength of the dissipative interaction between the particles of the system. σ represents the noise intensity. They are related via the fluctuation-dissipation theorem[3,7]

$$\sigma^2 = 2\gamma k_B T, \tag{S7}$$

$$\omega^D(r_{ij}) = [\omega^R(r_{ij})]^2 = \begin{cases} \left(1 - \frac{r_{ij}}{r_c}\right)^2 & r_{ij} < r_c, \\ 0 & r_{ij} \geq r_c. \end{cases} \tag{S8}$$

where k_B is the Boltzmann constant and T is the absolute temperature of the system. In Eq. (S6), ξ_{ij} is a random number with zero mean and unit variance. These forces are of short-range with a fixed cutoff distance r_c. For simplicity, we choose the bead mass m, the cutoff radius r_c, and the energy k_BT as units: $m = r_c = k_BT = 1$. Therefore, the relevant time unit is also $\tau = 1$. Unless specified otherwise, all dimensional values are given in DPD reduced units. The Groot-Warren-velocity-Verlet algorithm[3,8] is used here to integrate Newton's equations of motion. In our simulations, we set the number density of the system $\rho = 3$, and the time step $\Delta t = 0.02$.

To simplify our model, we omit the complex details of a specific enzyme, such as its surface charge or amino acid distribution. As illustrated in Scheme 1 in the paper, we represent the enzyme as a spherical particle (O/N, golden yellow), as most enzymes adopt a globular protein structure. The diameter of globular protein molecules usually ranges from a few nanometers to several tens of nanometers[9–11]. Based on this, we chose a moderate value (r = 5.0 r_c, the radius of the sphere, with $r_c \cong 1.0$ nm) as our representing size parameter for globule proteins. The sphere is constructed via the geodesic subdivision method, which developed by our group in early studies[12–15]. In practice, to make sure the sphere is impenetrable, we generate an outer shell (O) and an inner shell (N) with slight radius difference (e.g., $R_{outer} - R_{inner} = 0.5r_c$). In each shell, the surface density of the sphere-type beads is 3.0. As a result, the number of beads used in modeling outer and inner shell is 942 and 763, respectively. In our simulations, all beads of the sphere are treated together as a rigid body. The quaternion method is applied on the beads of the same sphere to constrain mutual movement of the sphere-type beads in the same sphere. With this generalized model, it is easy to incorporate the chemical and structural characteristics of a specific enzyme within the simulation framework outlined here. The monomer we designed is composed of a two-part functional group containing an adsorption bead and a polymerization bead. In the simulation, we coarse-grained them into beads A (dark blue) and B (light blue). There is a strong attraction between bead A and the sphere, which means that bead A is adsorbed on the surface of the sphere relying on the physical adsorption property, rather than reacting directly with the sphere. Bead B is a polymerization bead with hydrophobic properties, which can be interpreted as a group containing an unsaturated double bond that can participate in a polymerization reaction. The pink region represents the crosslinker beads (C) after coarse-graining. Initiators (I) and solvents (W) are randomly placed within the simulation box but are not depicted in Scheme 1.

Additionally, to facilitate delivery of polymer nanocapsules into cells, we introduce a hydrophilic chain (E in Scheme 1, light yellow)

modified with a polymerizable bead (C). This modification is expected to improve the circulation of the nanocapsules in human body, thereby enhancing their functionality[16,17]. In our simulations, following free radical polymerization, a thin protective polymer shell forms on the surface of the sphere, resulting in the polymer nanocapsule, which will be referred to as n(sphere) hereafter.

In our simulations, the repulsive parameters α are determined based on the properties and interactions of different types of beads. Generally, for any two beads of the same type, we set the repulsion parameter to $\alpha_{ii} = 25.0$. Hence, α_{ij} will be larger than 25.0 when the bead-bead repulsion is stronger. Typically, the external amino acid residues of proteins exposed to the solvent are hydrophilic, while the nonpolar residues within the protein's tertiary structure are mostly hydrophobic. To simplify the model, we use the DPD repulsion parameters to represent the amphiphilic character of the protein by defining simple interactions between the solvent and the various types of beads in the nanoparticles. In practice, we set the hydrophilic interaction parameter between the outer layer bead (O) and the solvent to $\alpha_{WO} = 25.0$, and the hydrophobic interaction between the inner layer bead (N) and the solvent to $\alpha_{WN} = 45.0$. Additionally, to prevent other besds from penetrating the nanoparticle body, we set the repulsive parameter for the N-type bead interactions (α_{Nj}, where j refers to the other bead types in the solvent environment) to a higher value ($\alpha_{Nj} = 45.0$).

Literature indicates that monomers used to fabricate enzyme-polymer nanocapsules are rich in functional groups, such as amide, acyl oxygen, amino, and hydroxyl, which can form hydrogen bonds with proteins[18,19]. We initially estimate the solubility parameters of various simple molecules containing these groups. From this, we can calculate the Flory-Huggins interaction parameters χ_{ij}[20,21] and note that the estimated values show minimal variation. The interaction parameters for the simulation can be calculated using the relationship $\alpha_{ij} = \alpha_{ii} + 3.27\ \chi_{ij}$[22]. To maintain simplicity, we implement uniform and moderate values, setting $\alpha_{AB} = \alpha_{AC} = \alpha_{AE} = \alpha_{BE} = \alpha_{CE} = 26.5$ and $\alpha_{WA} = \alpha_{WB} = \alpha_{WC} = 28.0$. Considering the interactions between protein and monomer, we assign a weakly repulsive parameter for the interaction between the outer bead (O) and the monomer beads (B, C, E), setting $\alpha_{OB} = \alpha_{OC} = \alpha_{OE} = 28.0$.

Ultimately, we determine the interaction parameters in this study, as detailed in Table S1. In addition, we calibrate the Flory-Huggins parameters between different types of beads, as detailed in Table S2.

Table S1 DPD interaction parameters used in the simulations

$\alpha_{ij}(k_BT/r_c)$	A	B	C	E	W	O	N	I
A	25.0	26.5	26.5	26.5	28.0	α_{OA}	45.0	25.0
B		25.0	25.0	26.5	28.0	28.0	45.0	25.0
C			25.0	26.5	28.0	28.0	45.0	25.0
E				25.0	25.0	28.0	45.0	25.0
W					25.0	25.0	45.0	25.0
O						25.0	25.0	25.0
N							25.0	45.0
I								25.0

When modeling the monomer and the polymer, the bonds between beads in the molecules are described via the harmonic spring potential,

$$U_{bond}(r) = \frac{1}{2}k_b(r - r_0)^2, \tag{S9}$$

where k_b is the spring constant, and r_0 is the equilibrium bond length. In our simulations, we set generic bond constant and equilibrium bond length, where $k_b = 4.0$ and $r_0 = 0.85\ r_c$ [23–25]. The flexibility of the polymer chains is adjusted by incorporating the bending angle potential of the neighboring three beads,

$$U_{angle} = k_{angle}[1 - \cos(\theta - \theta_0)], \tag{S10}$$

where θ_0 is the equilibrium angle with $\theta_0 = 150$ and k_{angle} is the angle force constant. In our simulations, we adjust the value of k_{angle} to vary from 0.0 (fully flexible chains) to 16.0 (semi-rigid chains)[24,26] to investigate the effect of the chain rigidity on the structure of the nanocapsules. In other simulations, k_{angle} is set to 4.0 (flexible chains) unless otherwise stated.

Table S2 Flory–Huggins χ-parameters used in the simulations

$\chi_{ij}(k_BT/r_c)$	A	B	C	E	W	O	N	I
A	0.0	0.5	0.5	0.5	0.9	χ_{OA}	6.1	0.0
B		0.0	0.0	0.5	0.9	0.9	6.1	0.0
C			0.0	0.5	0.9	0.9	6.1	0.0
E				0.0	0.0	0.9	6.1	0.0
W					0.0	0.0	6.1	0.0
O						0.0	0.0	0.0
N							0.0	6.1
I								0.0

To evaluate polymer chain flexibility in our system, we calculated the persistence length (l_p) of 100 polymer chains with a degree of polymerization of 100 in θ consition. We evaluated this by analyzing the chain segment orientation correlation, while the intra-chain orientation correlation can be obtained by calculating the bond-bond correlation function. The formula is as follows: $\langle \cos\theta(s)\rangle = \frac{\langle \boldsymbol{b}_j \cdot \boldsymbol{b}_{j+s}\rangle}{l_b^2}$, where s is the curvilinear (chemical) distance between the jth bead and the $(j+s)^{th}$ bead along the same chain, and l_b is the average bond length. The so-called persistence length (l_p), which is also one of the microscopic parameters characterizing the intrachain length scales and the chain stiffness, can be derived from the formula as $\langle \cos\theta(s)\rangle = \exp\left(-sl_b/l_p\right)$[27]. We set the average chain length (l_b) of the polymer chains in our simulations, equal to 0.85 r_c (r_c is approximately 1 nm), and after fitting the curves we calculated l_p to be approximately 1.56 nm (Figure S17). The studies[28–30] show that the persistence lengths of the flexible chains obtained from the characterization of the experimental system are in the range of 0.3-2 nm, which is caused by the localized flexibility dominated by the single-bond rotational potentials. For example: poly(1-adamantyl acrylate) (persistence length ∼1.2 nm)[29], poly(methyl methacrylate) (persistence length ∼2.0 nm)[29], polystyrene (persistence length ∼1.2 nm)[28], polyethylene (persistence length ∼0.8 nm)[30]. The persistence lengths of the polymer chains of our system fall into the flexible chain interval, which is consistent with our expectations and matches the conclusions of previous simulation work[24]. Based on this, we can judge the flexibility of the polymer chains by using the persistence length as a bridge between the simulation and the experimental system.

In our simulations, we adopt a reaction model to describe the polymerization process by introducing the concept of reaction probability (P_r)[31,32]. During the reaction, when the initiator encounters multiple free monomers, it randomly selects a monomer within the capture radius (for simplicity, set to equal to the cutoff radius) as the target for the reaction. A random number (P) is then generated, and by comparing it to a predetermined reaction probability (P_r), we determine whether the reacting object will be connected to the active end or not. When P < P_r, the reacting object participates in the reaction and serves as the growth center for the polymer chain, facilitating further chain growth. This reaction probability can effectively represent the reaction rate constant of the system. In order to better characterize the relationship between the polymerization probability and the polymerization groups during the experiments, we have converted them according to the following mapping. According to our previous work[24,33], the reaction probability (P_r) that represents the reaction rate of the system can be expressed as an Arrhenius-type equation, P_r = Aexp(-E_a/(k_BT)), where A is a modifying factor, which can be calibrated through a specific reaction. E_a is the activation energy of the reaction, k_B is the Boltzmann constant, and T is the absolute temperature. In Ref. 29, the authors used a general free radical polymerization activation energy value at 50°C to estimate the modifying factor A, which was calculated to be A = 2.2×10^5. We used this value to estimate the activation energy of our system. When P_r = 0.005, the calculated E_a was about 43 kJ/mol. For experimental systems, this value is mostly common for the activation energy for the polymerization of vinyl-based monomers[34,35].

To characterize the structural and compositional properties of as-formed polymer nanocapsule, we calculate several key physical quantities in the simulations. The relative shape anisotropy (κ^2) of the nanocapsule shell structure is used to assess its regularity. To obtain a more accurate measurement of the shell structure, we exclude the contribution from the hydrophilic chains. The gyration tensor of the entire polymer nanocapsule is first calculated, and its eigenvalues λ_1, λ_2 and λ_3 are obtained in descending order (i.e., $\lambda_1 \geq \lambda_2 \geq \lambda_3$)[36,37]. The relative shape anisotropy is then calculated as follows:

$$\kappa^2 = 1 - 3\frac{\lambda_1\lambda_2 + \lambda_2\lambda_3 + \lambda_3\lambda_1}{(\lambda_1+\lambda_2+\lambda_3)^2}. \tag{S11}$$

This value reflects the symmetry of the nanocapsule shell, with κ^2 ranging from 0 to 1. A value of 1 corresponds to an ideal linear conformation, while a value approaching 0 indicates highly symmetric spherical conformations[36–38]. To compare the adsorption capacity of monomers to the spherical nanoparticle under different conditions, we define the adsorption density (ρ_{ads}):

$$\rho_{ads} = N_{particles}/V, \tag{S12}$$

where $N_{particles}$ represents the number of initiators (I), reactive monomers (A-B), and crosslinkers (C-C) within a thin shell with a thickness dr (=1.0), measured from the surface of the spherical particle, and V represents the volume of this thin shell.

To calculate the thickness of n(sphere)'s polymer shell, we take the center of the sphere as the origin and count the density distribution curve of the polymer shell in the radial direction. We take 0.02 as the domain value where the density profile decreases, and 90% of the difference in the horizontal coordinates of this value is recorded as the thickness. To assess the impact of the size of catalytic substrates on their ability to interact with real enzymes, we calculate the solvent accessible surface area (SASA) of the nanoparticle surface[39]. This is achieved by moving a probe bead (such as a water molecule) around the van der Waals surface of the n(sphere), marking the contact points between the probe and n(sphere). We then compute the total area of all marked points to obtain the SASA value. In our statistical analysis of the simulation data, we use the SASA of the n(sphere) surface as a standard for comparison among different structures.

To evaluate the utilization of reactants in the system, we define the degree of bead participation in the polymerization reaction (η_{react}):

$$\eta_{react} = N_{shell}/N_{all}, \tag{S13}$$

where N_{shell} represents the number of beads involved in forming the polymer shell, which includes all reactive monomers (A-B), crosslinkers (C-C), initiators (I), and the modified hydrophilic chain end groups. N_{all} is the total number of beads in the initial state of the system, excluding water beads.

Furthermore, to verify the effect of sphere size on the conformation of the nanocapsules, we altered the size of the sphere to 3.0 r_c, we could similarly obtain structurally regular polymer nanocapsules (Figure S16). So, changing the size of the sphere does not affect the final result. However, the number of hydrophilic chains also affects the polymer shell layer's regularity. The concentration of hydrophilic chains in the system needs to be pre-tuned by adjusting the sphere size. As the size increases, the concentration can be increased appropriately.

To design a functional monomer for nanocapsule formation, we considered two distinct components in one monomer, i.e., in the model, an adsorbable bead A and a polymerizable bead B, which serve as the anchoring unit and the polymerization unit, respectively. For the successful formation of a polymer shell on a specific protein surface, effective anchoring of bead A is essential. Therefore, we first examined the chemical composition of the adsorbable bead A independently. Using bovine serum albumin (BSA) in neutral aqueous solution as a model, we analyzed the chemical/structural requirements for bead A in the monomer. The chemical environment of BSA's surface is known to be rich in polar and charged functional groups-such as carboxyl, amino, and hydroxyl groups[40,41]. Literature also reports that in dissipative particle dynamics (DPD) simulations, amide- and alcohol-based beads exhibit strong non-bonded interactions in water, with interaction parameters around 10[42]. To reflect these interactions, we selected acetamide as the representative functional group for bead A due to its compatible polarity and hydrogen bonding capacity. We then introduced various modifications to acetamide and assessed their protein-binding ability using all-atom molecular dynamics (AAMD) simulations. As a baseline, we calculated the solvent-accessible surface area (SASA) of each amino acid residue in pure BSA under neutral aqueous conditions. The mean SASA values for each residue type were used to estimate their exposure on the protein surface. Our results revealed that hydrophilic residues such as lysine, glutamic acid, aspartic acid, and alanine are abundantly present on the BSA surface (Figure S18). We quantified their binding affinities by calculating the residence time of each molecule's adsorptive bead in contact with BSA residues when we added equal concentrations of different small molecules into the system. The residence time of the adsorbable molecule (bead A) is defined as the average duration of the molecule's sustained interaction with the target residue, providing insight into the affinity between our constructed adsorbable beads and the BSA protein residues. The residence time was calculated from the normalized survival time correlation function $\sigma(t)$:

$$\sigma(t) = \frac{1}{T_f - t} \sum_{\nu=0}^{T_f - t} \left(\frac{1}{N_j} \sum_{j=1}^{N} \tilde{n_j}(\nu, \nu + t) \right), \tag{S14}$$

where T_f is the total simulation time, and N_j is the total number of effective contacts within a dual-cutoff distance of 0.25 nm and 0.4 nm. The duration of a contact is defined as the time between the frame when the small molecule with adsorption capacity moves closer than the lower cutoff and the frame when it moves beyond the upper cutoff. $\tilde{n_j}(\nu, \nu+t)$ is assigned a value of 1.0 if the small molecule continuously interacts with residues from time ν to time ν + t, and 0 otherwise. The function $\sigma(t)$ was calculated for each frame from ν to T_f and normalized by dividing by $\sigma(0)$. The normalized survival time correlation function for constructed adsorbable molecule interactions with each residue was then fitted to a biexponential function to extract the long and short decay rates of molecule relaxation:

$$\sigma(t) \sim Ae^{-k_1 t} + Be^{-k_2 t}. \tag{S15}$$

The constraint for fitting was set to$k_1 \leq k_2$. The residence time τ corresponding to each molecule was then obtained from $\tau = 1/k_1$.

The representative results of the residence time of small molecules on the protein surface are shown in Figure S19. Additionally, we found that a residence time of 1.0 nanoseconds or greater is defined as indicative of stable adsorption[43]. To identify potential adsorption sites, we assessed the solvent exposure of residues based on their solvent-accessible surface area. Residues with an actual SASA greater than 30% of their theoretical maximum were classified as solvent-exposed and thus available for molecular interaction[44,45].

Figure S19 (a)-(e) show the top 20 residues with the longest average residence times for each small molecule, revealing significant differences in adsorption patterns. Functional groups such as -CH_3, -OH, -NH_2, and -COOH drive interactions through hydrogen bonding, electrostatic forces, hydrophobic contacts, and *pi-pi* stacking. These diverse and distributed binding mechanisms enable certain molecules to form continuous adsorption shells, offering a structural scaffold for in situ radical polymerization and nanocapsule formation. Figure S19 (f) summarizes the percentage of stable adsorption events occurring on solvent-exposed residues. The data demonstrate a strong correlation between adsorption stability and the chemical nature of substituents. Among the tested molecules, NH_2COCH_2COOH exhibits the highest affinity, with residence times exceeding 100 ns at several residues. This is attributed to its -NH_2/-COOH groups forming multiple hydrogen bonds and salt bridges with both acidic and basic residues. Similar behavior has been reported for carboxylic acid-containing drugs binding to BSA[46]. $NH_2COCH_2NH_2$ also shows strong binding, facilitated by dual -NH_2 groups that interact with ASP, GLU, and TYR. In contrast, NH_2COCH_2OH, relying solely on hydrogen bonding, exhibits a moderate adsorption. Esterification in $NH_2COCH_2COOCH_3$ neutralizes the carboxylate charge, weakening both ionic and hydrogen bonding interactions. The least polar compound, NH_2COCH_3, interacts mainly through weak van der Waals forces and hydrogen bonding interactions, resulting in the lowest adsorption stability.

Examples of the chemical components and properties of polymerization groups are as follows: The optimal chain rigidity for forming a well-structured nano-shell lies in a semi-flexible regime, i.e., the polymer chains should not be completely flexible but must retain sufficient conformational adaptability. We calculated the persistence length (l_p) of polymer chains (the number of chains and the degree of polymerization are set to 100) under θ-solvent conditions to evaluate chain stiffness[27]. After fitting the curve, we calculated l_p to be approximately 1.56 nm (Figure S16). The references[28–30] showed that the persistence lengths of flexible chains obtained in experiments are in the range of 0.3-2.0 nm. Additionally, the activation energy Ea of the reaction is predicted to be about 43 kJ/mol. For experimental systems, this value is mostly common for the activation energy for the polymerization of vinyl-based monomers[34,35]. Combining these facts, we can use simple propylene-based polymerizable monomers as the polymerization bead B. Strategically, we propose to use polymerizable monomers with small, sterically non-intrusive adsorbing groups. These groups contribute minimally to chain rigidity, ensuring that the resulting vinyl polymer chains stay within the l_p range. Moreover, the polymerization reaction rate can be regulated by adjusting the reaction temperature, which in turn has a good regulation of the polymerization reaction. Therefore, it is necessary to make some initial temperature attempts during the actual preparation process in experiments and fine-tune the reaction temperature by observing the generated structure of the nanocapsules to achieve the optimal polymerization condition.

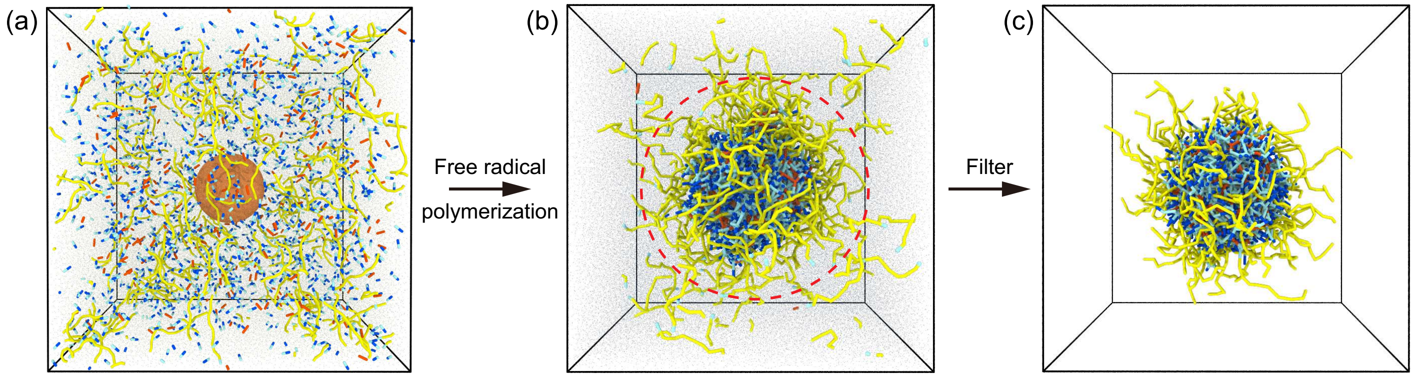

Figure S1 Synthesis process of n(sphere) in our simulation. Snapshots show the main view of the initial state (a), post-polymerization (b), and the system after removing non-participating elements (c). The molar ratio of monomers, crosslinkers, and initiators in the above reaction is 75:14:1, namely $n_{(M)}$: $n_{(C)}$: $n_{(I)}$ = 75:14:1. Simulation parameters are established with α_{OA} = 6.0 and P_r = 0.005. Visual representations employ the following color : central sphere (O, gold), crosslinkers (C-C, pink), monomer adsorbable beads (A, dark blue), polymerizable beads (B, light blue), and hydrophilic chains (E_{10}, yellow). Solvent molecules are omitted from visualization for clarity.

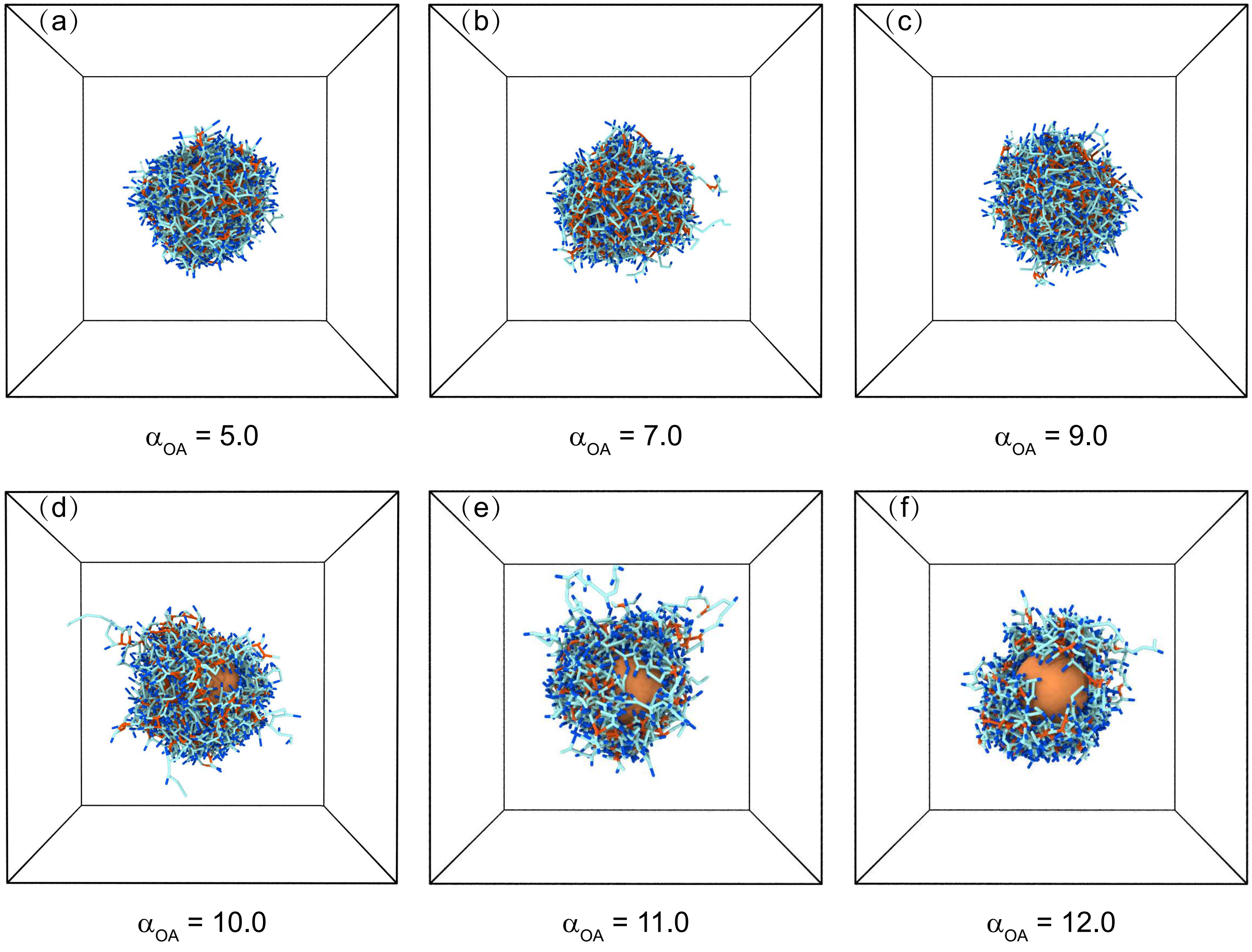

Figure S2 Snapshots of n(sphere) at different adsorption capacities (α_{OA}) with the same feeding ratio as Figure S1. The reaction probability is equal to 0.005. Hydrophilic chains and solvents are not shown. Color scheme is the same as in Figure S1: central nanosphere (gold), crosslinkers (pink), adsorption beads (dark blue), polymerization beads (light blue).

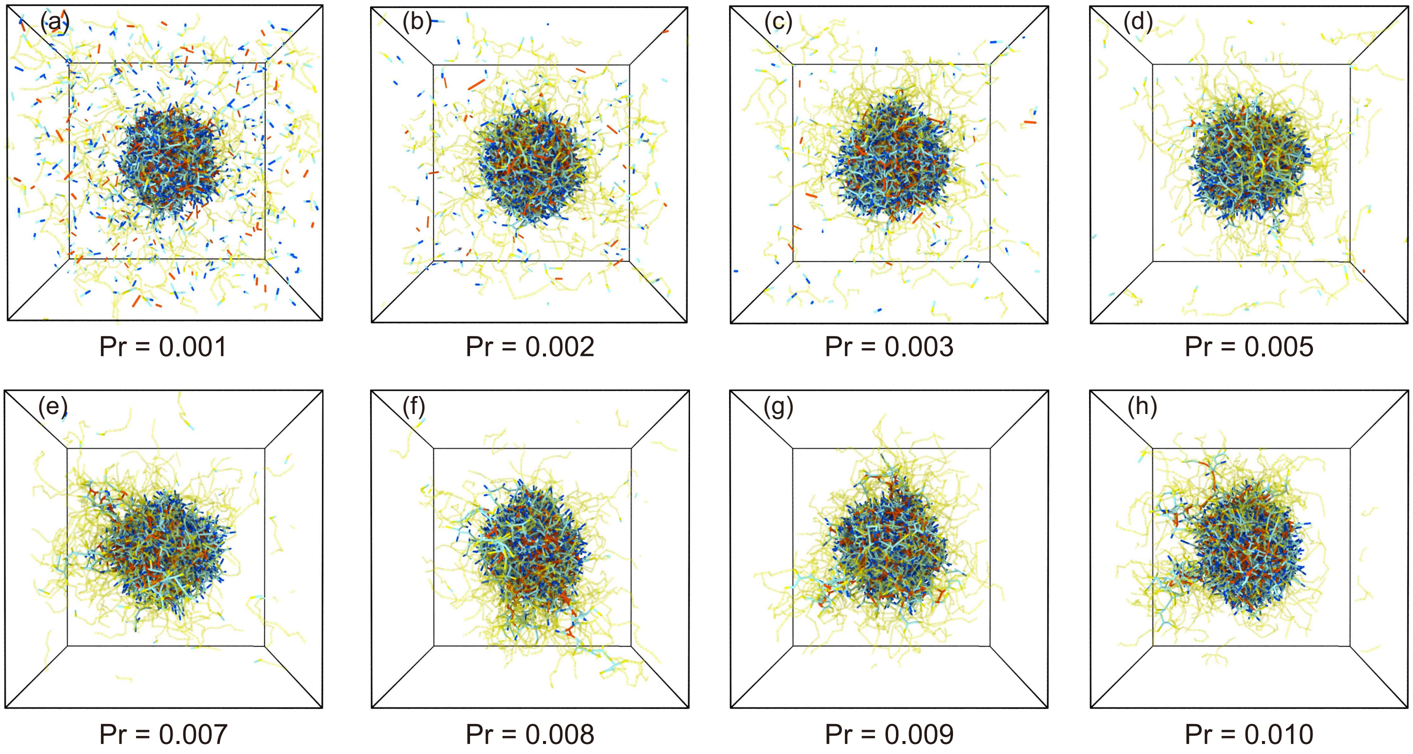

Figure S3 Snapshots of n(sphere) at different reaction probability (P_r) with the same feeding ratio as Figure S1. In the simulation snapshot, solvent is not shown for clarity and the color settings are the same as those in Figure S1. All simulations were conducted under standardized conditions (α_{OA} = 6.0).

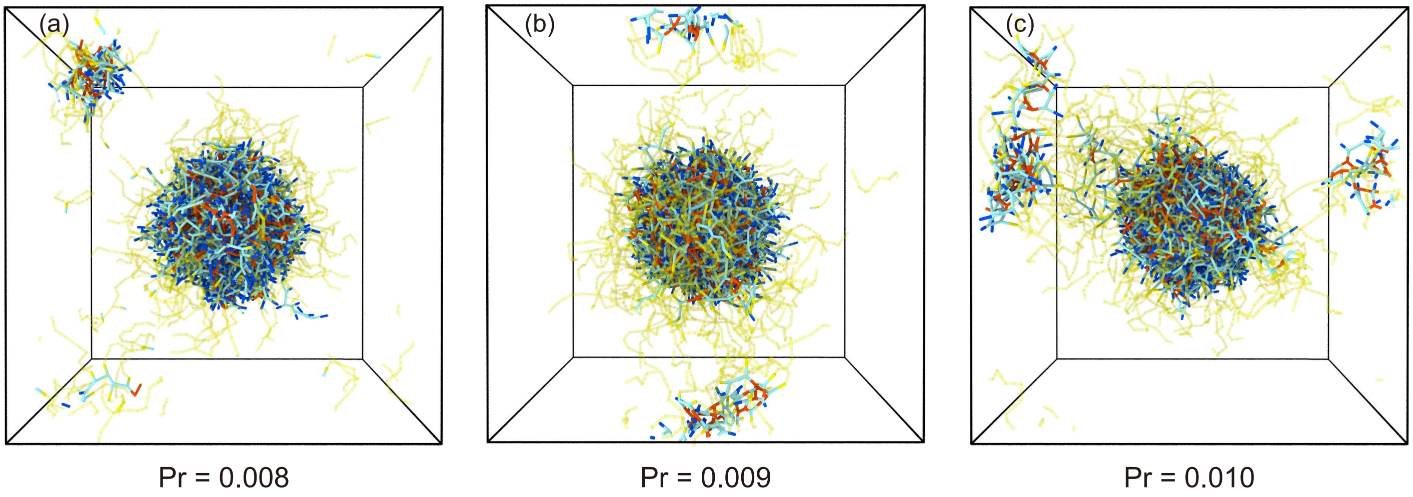

Figure S4 Snapshots of n(sphere) under a higher reaction probability (P_r) with the same feeding ratio as Figure S1. In the simulation snapshot, solvent is not shown for clarity and the color settings are the same as those in Figure S1. All simulations were conducted under standardized conditions (α_{OA} = 6.0).

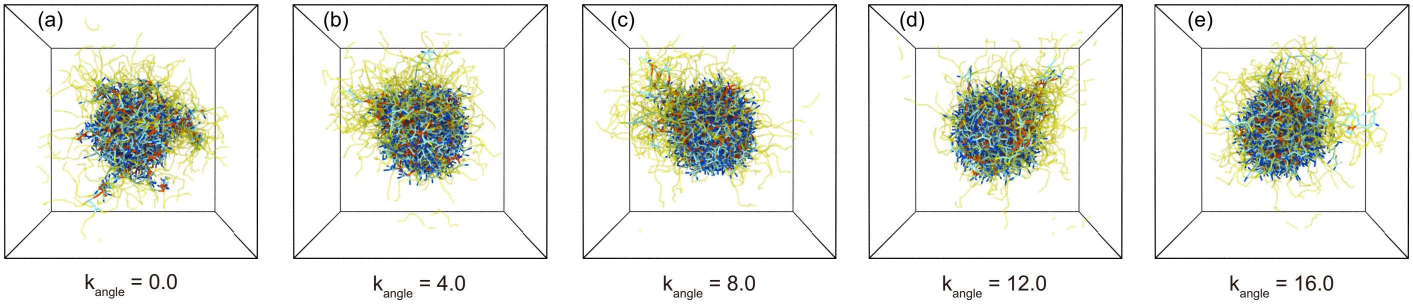

Figure S5 Snapshots of n(sphere) at different k_{angle} values. The molar ratio of monomers, crosslinkers, and initiators in the above reaction is 75:14:1, namely $n_{(M)}$: $n_{(C)}$: $n_{(I)}$ = 75:14:1. All simulations were conducted under standardized conditions (α_{OA} = 6.0, P_r = 0.005). In the simulation snapshot, solvent is not shown for clarity and the color settings are the same as those in Figure S1.

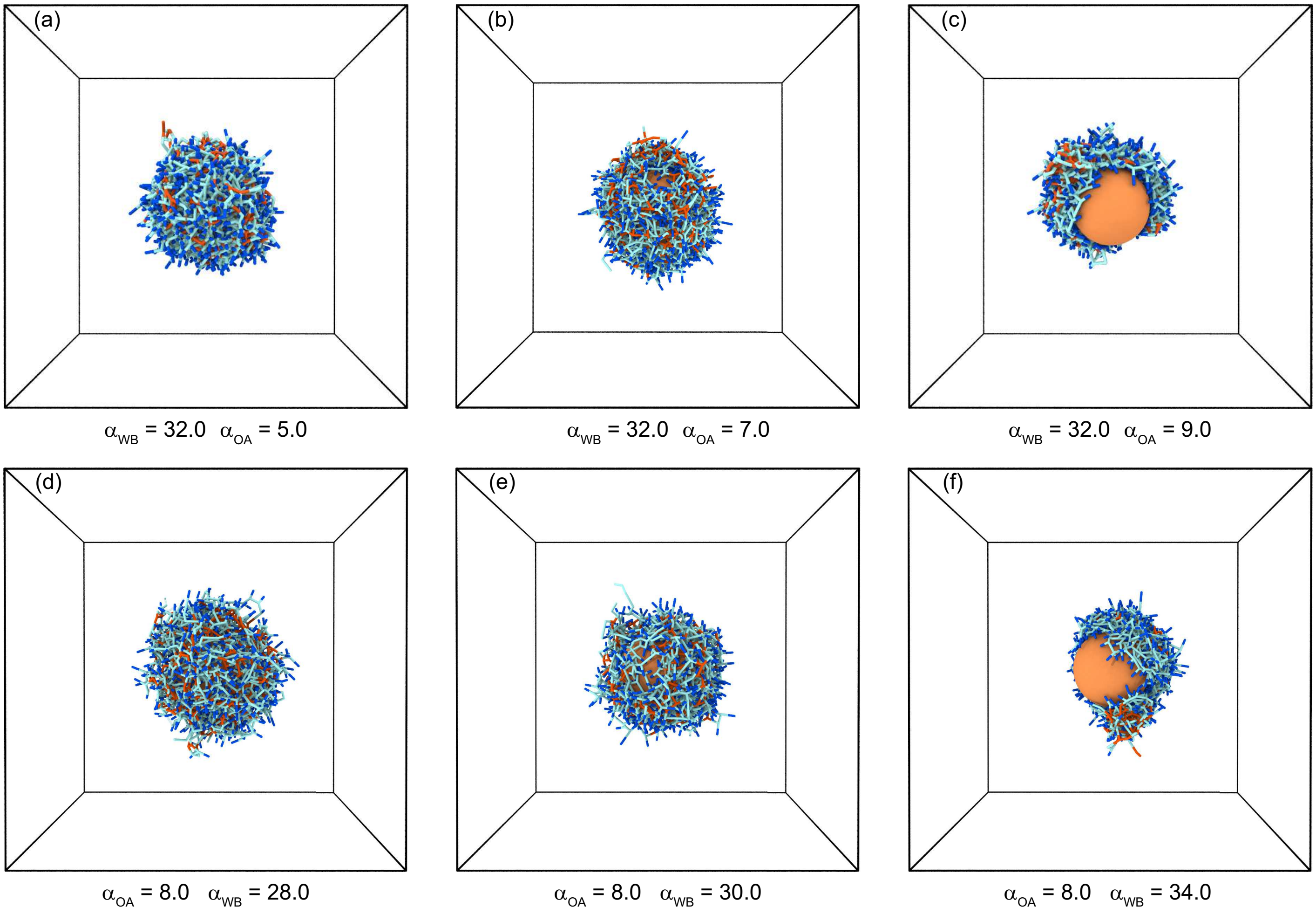

Figure S6 Snapshots of n(sphere) at different adsorption capacities (α_{OA}) and hydrophobic strength (α_{WB}) when P_r is equal to 0.005. Figures (a), (b) and (c) show simulation snapshots with the same hydrophobic strength but different adsorption capacities. Figures (d), (e) and (f) illustrate simulation snapshots with the same adsorption capacity but varying hydrophobic strengths. Hydrophilic chains and solvents are not shown. Color scheme is the same as in Figure S1.

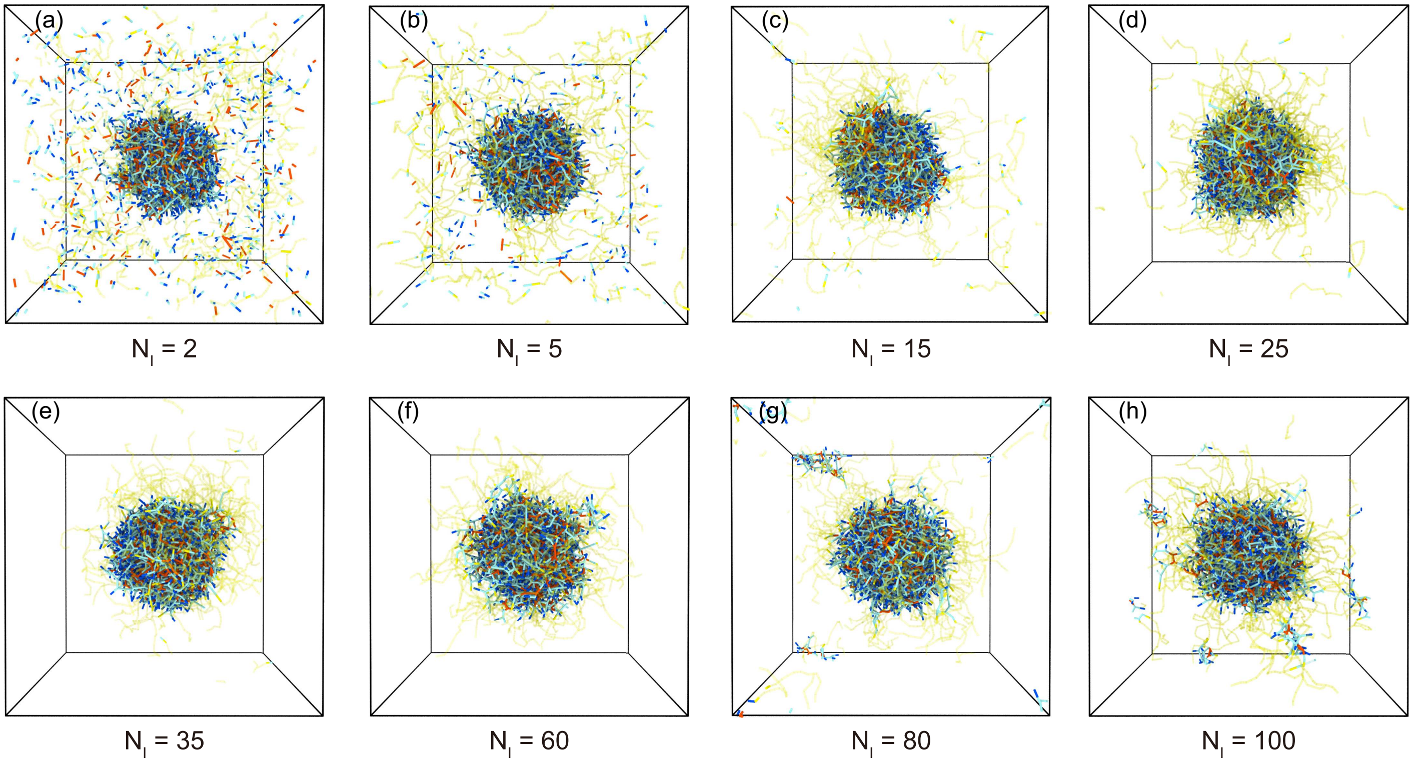

Figure S7 Snapshots of n(sphere) at different numbers of initiators when $N_M = 1500$, $N_C = 280$. All simulations were conducted under standardized conditions ($\alpha_{OA} = 6.0$, $P_r = 0.005$). In the simulation snapshot, solvent is not shown for clarity and the color settings are the same as those in Figure S1.

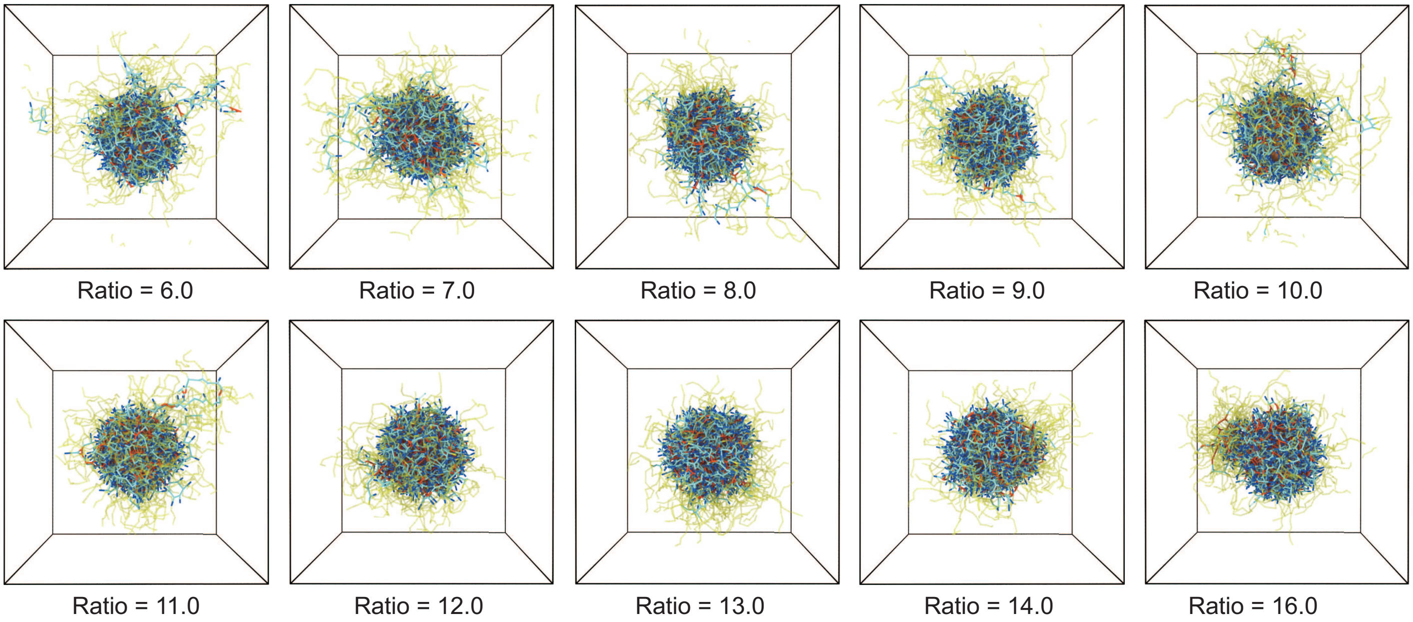

Figure S8 Snapshots of n(sphere) at different numbers of crosslinkers when $N_I = 20$. The ratio represents $n_{(C)}$: $n_{(I)}$. All simulations were conducted under standardized conditions ($\alpha_{OA} = 6.0$, $P_r = 0.005$). In the simulation snapshot, solvent is not shown for clarity and the color settings are the same as those in Figure S1.

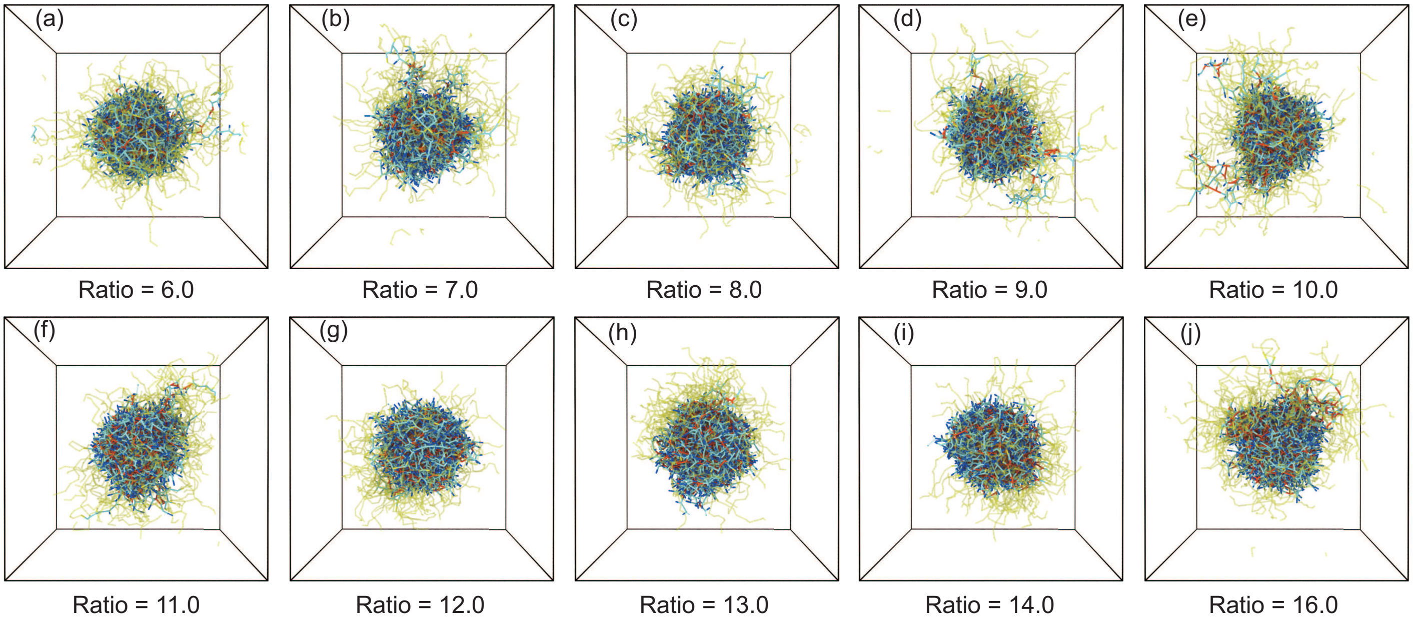

Figure S9 Snapshots of n(sphere) at different numbers of crosslinkers when $N_I = 25$. The ratio represents $n_{(C)}$: $n_{(I)}$. All simulations were conducted under standardized conditions ($\alpha_{OA} = 6.0$, $P_r = 0.005$). In the simulation snapshot, solvent is not shown for clarity and the color settings are the same as those in Figure S1.

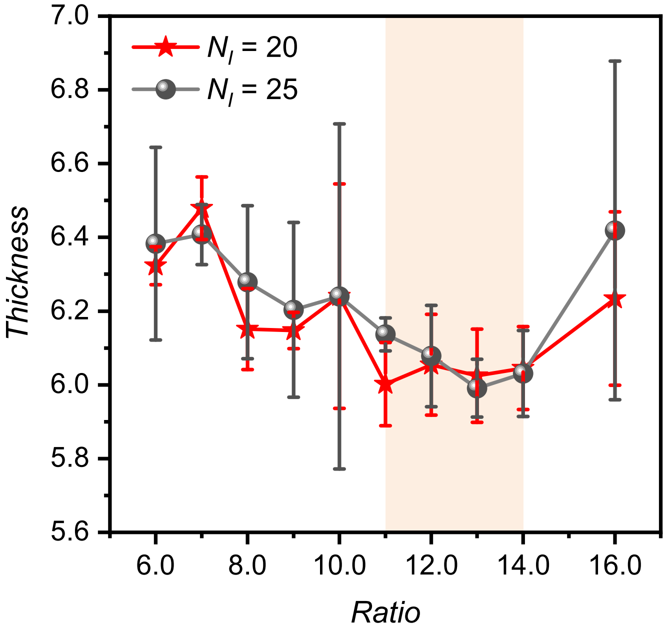

Figure S10 N(sphere) polymer shell thickness versus $n_{(C)}$: $n_{(I)}$ ratio at optimal initiator concentrations ($N_I = 20$, 25). It shows the influence of crosslinker concentration on the polymer shell thickness. All simulations were conducted under standardized conditions ($\alpha_{OA} = 6.0$, $P_r = 0.005$).

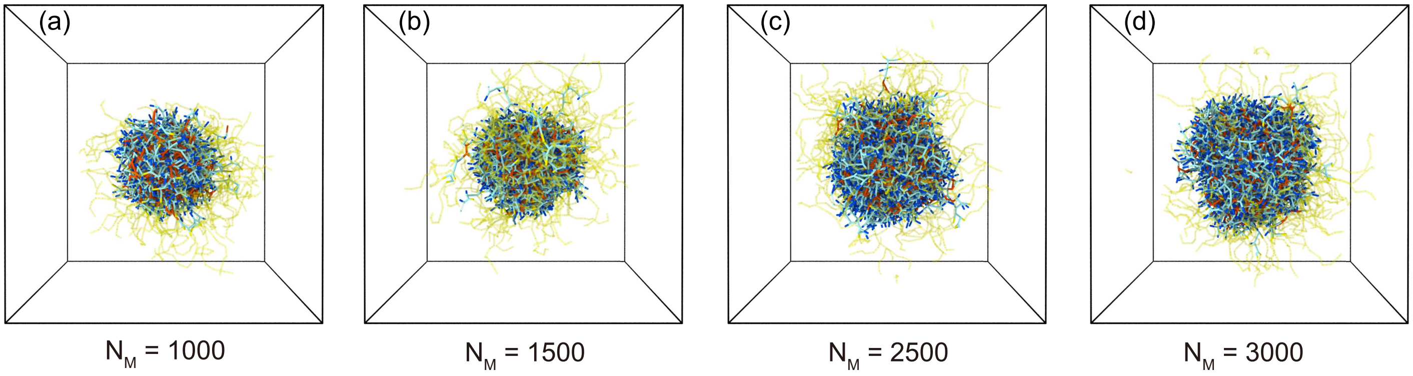

Figure S11 Snapshots of n(sphere) at different concentrations of monomers with a fixed feeding ratio. The molar ratio of monomers, crosslinkers, and initiators in the above reaction is 75:14:1, namely $n_{(M)}$: $n_{(C)}$: $n_{(I)}$ = 75:14:1. All simulations were conducted under standardized conditions (α_{OA} = 6.0, P_r = 0.005). In the simulation snapshot, solvent is not shown for clarity and the color settings are the same as those in Figure S1.

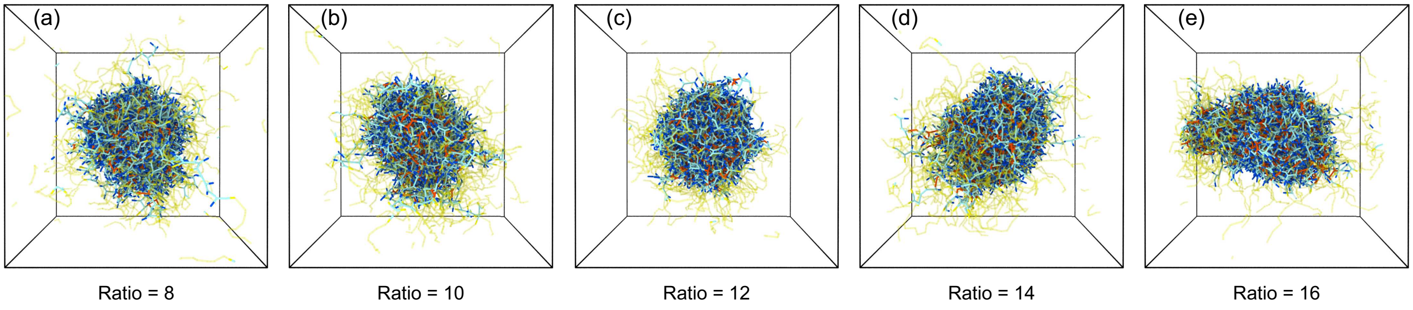

Figure S12 Snapshots of n(sphere) structures prepared with different crosslinker contents when α_{OA} = 6.0, P_r = 0.005, N_M=3000, and N_I=20. The ratio indicates $n_{(C)}$: $n_{(I)}$. In the simulation snapshot, solvent is not shown for clarity and the color settings are the same as those in Figure S1.

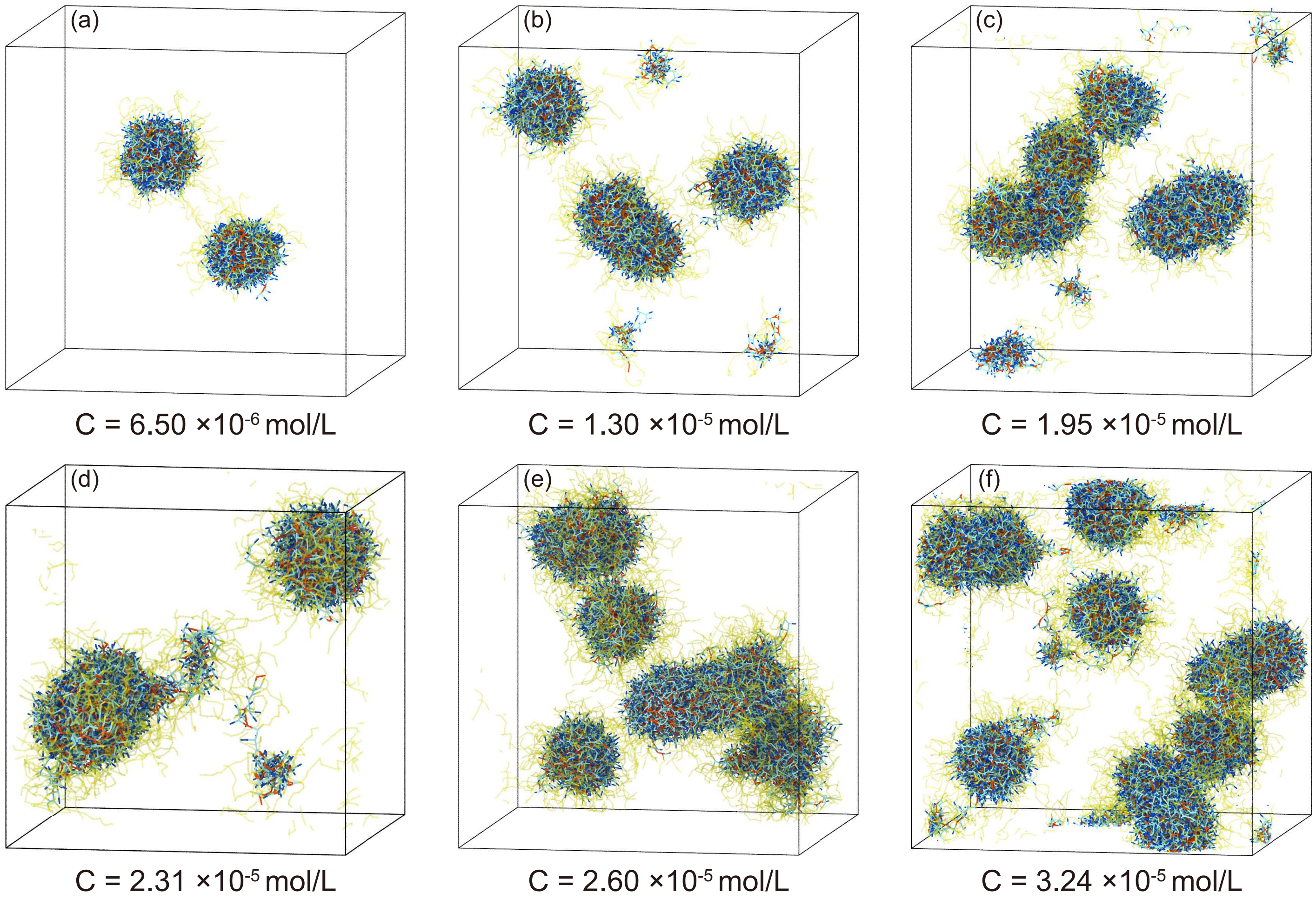

Figure S13 A side view of the nanocapsule-stabilized conformation of the multi-nanoparticle system from radical polymerization. The concentrations of the nanoparticle is shown under the snapshot. All simulations were conducted under standardized conditions (α_{OA} = 6.0, P_r = 0.005). In the simulation snapshot, solvent is not shown for clarity and the color settings are the same as those in Figure S1.

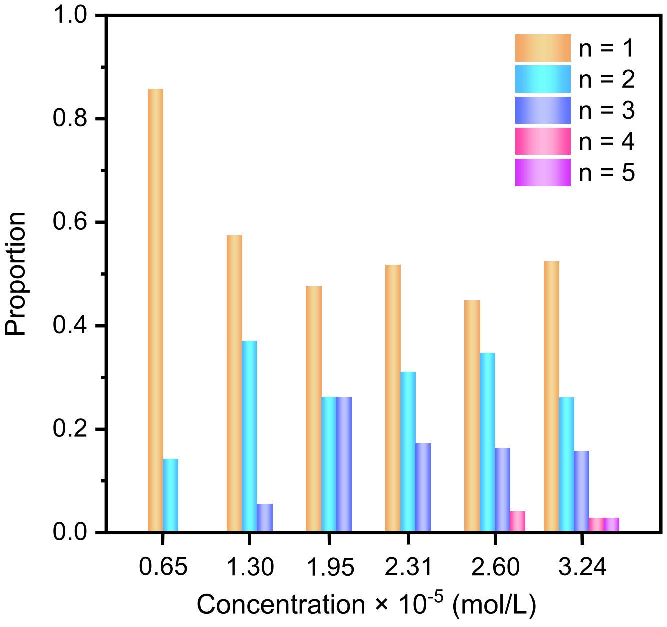

Figure S14 Polydispersity characterization of polymer nanocapsules at varying nanoparticle concentrations. The polydispersity arises from the differing amounts of the sphere within each nanocapsule, as indicated by n in the figure. The y-axis indicates the percentage of capsule structures corresponding to each dispersity level. All simulations were conducted under standardized conditions ($\alpha_{OA} = 6.0$, $P_r = 0.005$).

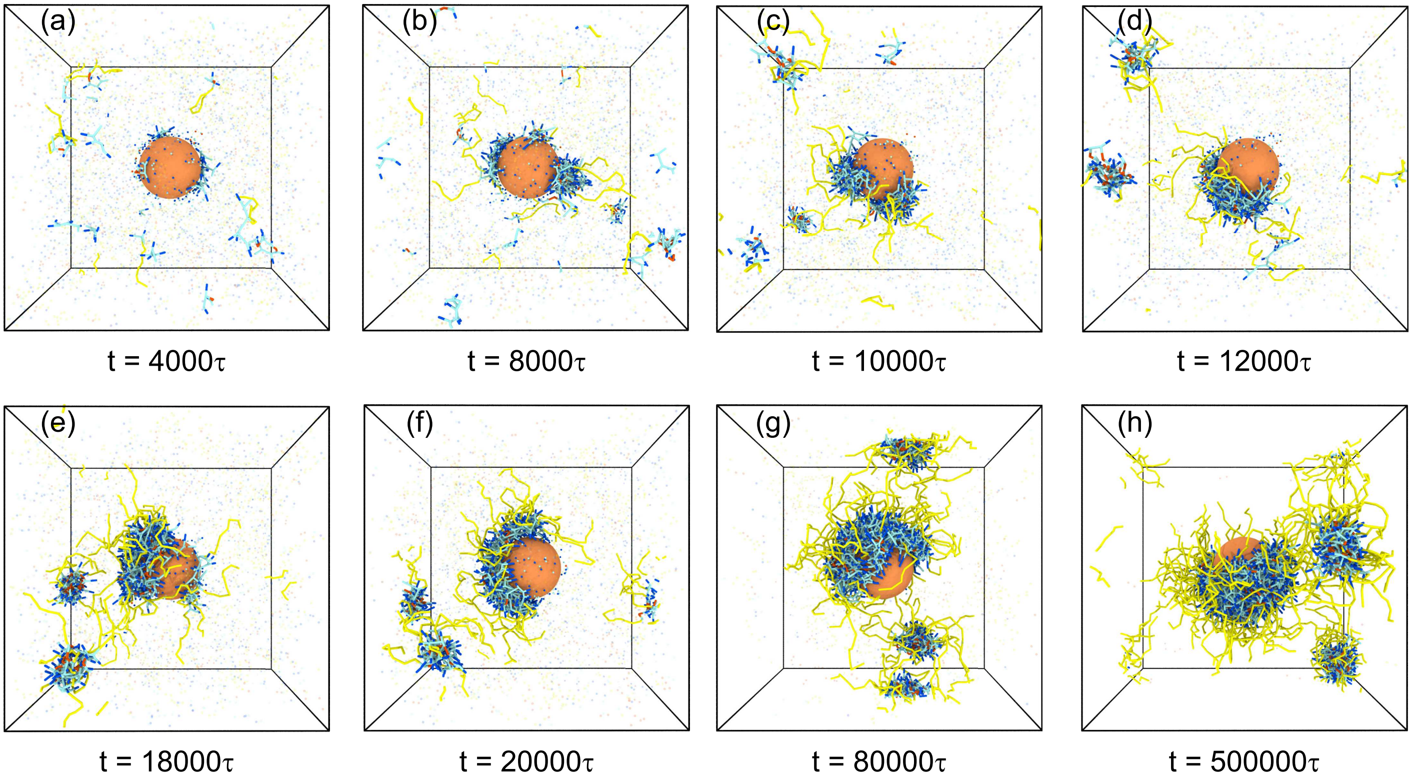

Figure S15 Time-resolved morphological evolution of n(sphere) assemblies at low adsorption and high hydrophobicity during polymerization. Reaction simulation parameters were maintained at $\alpha_{OA} = 8.0$, $\alpha_{WB} = 34.0$ and $P_r = 0.005$ throughout the process. Temporal progression is indicated below each snapshot. For visual clarity, solvent beads are omitted. The color settings are the same as those in Figure S1.

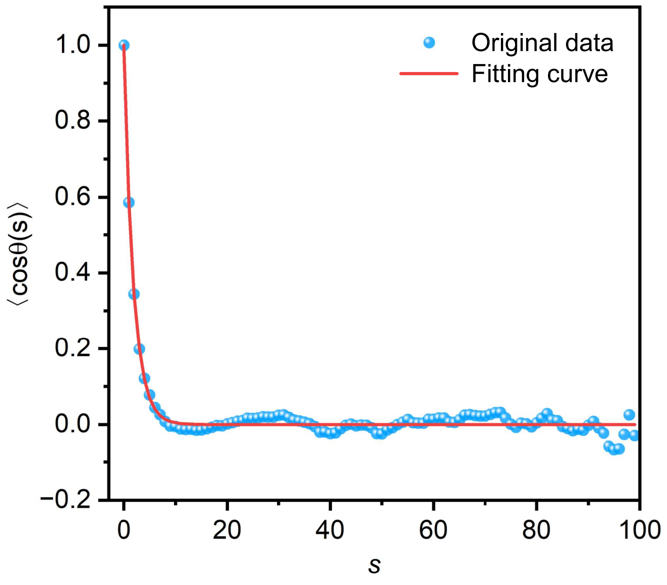

Figure S16 In DPD simulation, the bond-bond correlation function ($\langle \cos\theta(s) \rangle$) as a function of the chemical distance (s) between the beads. The results are shown for 100 polymer chains with a degree of polymerization of 100 in θ condition, with the blue spheres indicating the actual calculations and the red line indicating the fitted curve.

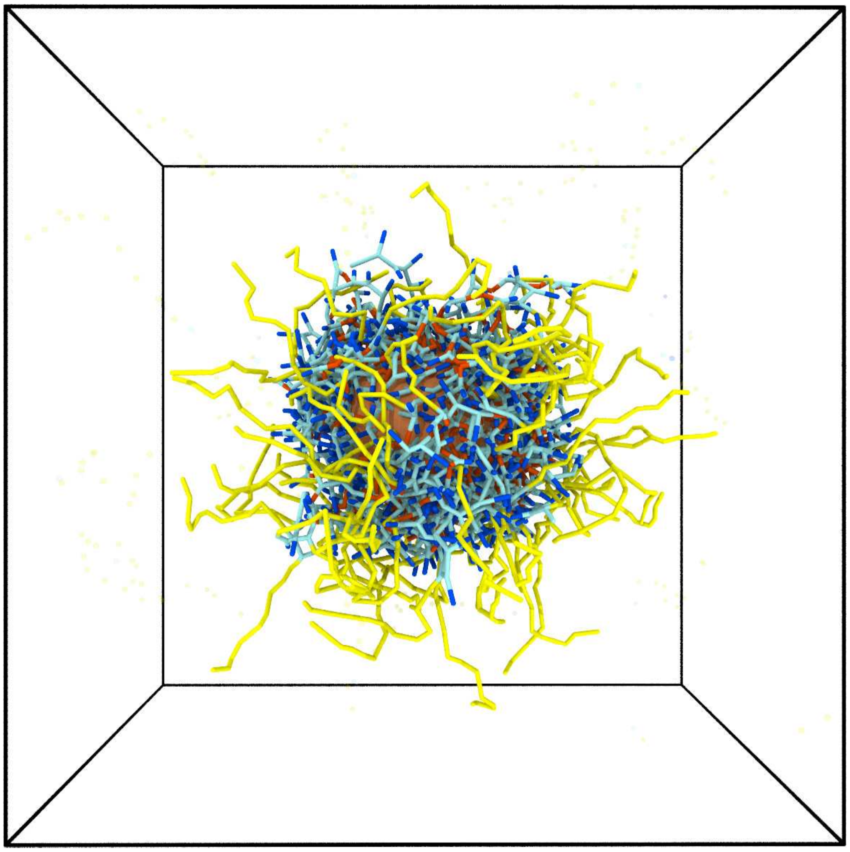

Figure S17 A snapshot shows the main view of the system after removing non-participating elements with a sphere radius of 3.0 r_c. The molar ratio of monomers, crosslinkers, and initiators in the above reaction is 75:14:1, namely $n_{(M)}$: $n_{(C)}$: $n_{(I)}$ = 75:14:1. The number of hydrophilic chains is 100. Simulation parameters are established with $\alpha_{OA} = 6.0$, $P_r = 0.005$). For visual clarity, solvent beads are omitted, and the particles that are not involved in the reaction are virtualised. The color settings are the same as those in Figure S1.

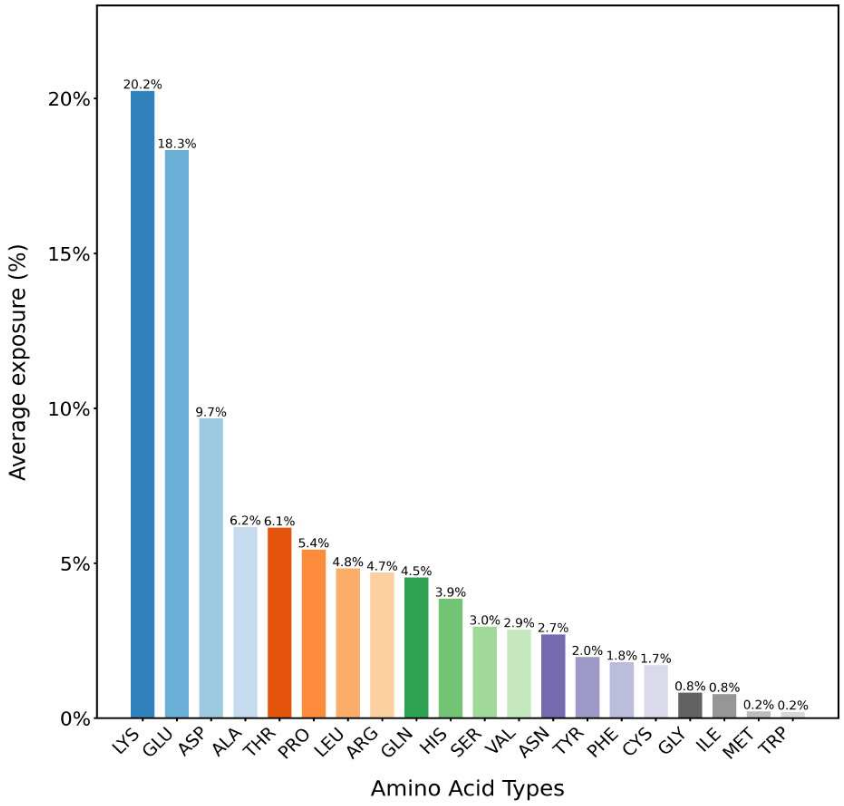

Figure S18 Average solvent exposure of amino acid residues of BSA protein when pH=7.0. The x-axis represents amino acid residue types, and the y-axis indicates the average percentage of solvent-accessible surface area (SASA), reflecting the degree of residue exposure. Values were averaged over 10,000 trajectory frames from the final 1.0 μs of the equilibrium AAMD simulation.

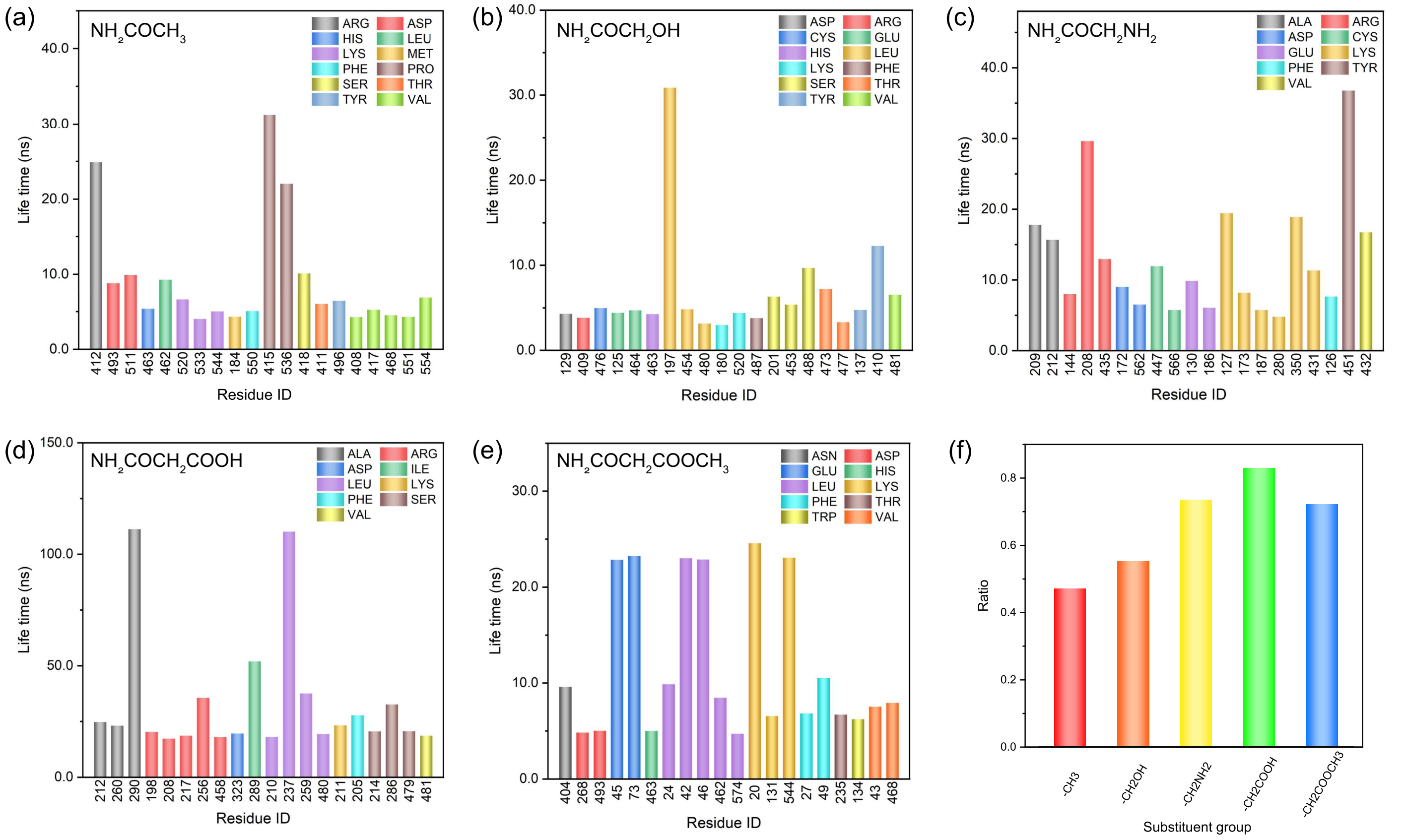

Figure S19 Residence time analysis and adsorption stability of five small molecules on the BSA surface. (a)-(e) Top 20 amino acid residues ranked by average residence time of five small molecules adsorbed on the BSA surface. Residence times were calculated relative to each residue, based on trajectory frames sampled every 0.1 ns over the final 300 ns of equilibration. Only residue names and indices with the residence are shown. (f) Proportion of stably adsorbed small molecules on surface residues of BSA exposed to the solvent environment, representing stabilized surface interaction ratios.

References

1 M. B. Liu, G. R. Liu, L. W. Zhou and J. Z. Chang, *Arch. Comput. Methods Eng.*, 2015, **22**, 529–556.

2 P. Español and P. B. Warren, *J. Phys. Chem.*, 2017, **146**, 150901.

3 R. D. Groot and P. B. Warren, *J. Chem. Phys.*, 1997, **107**, 4423–4435.

4 K. Yang and Y. Q. Ma, *Nat. Nanotechnol.*, 2010, **5**, 579–583.

5 R. K. Raya, M. Štěpánek, Z. Limpouchová, K. Procházka, M. Svoboda, M. Lísal, E. Pavlova, A. Skandalis and S. Pispas, *Macromolecules*, 2020, **53**, 6780–6795.

6 P. J. Hoogerbrugge and J. M. V. A. Koelman, *Europhys. Lett.*, 1992, **19**, 155–160.

7 P. Español and P. Warren, *Europhys. Lett.*, 1995, **30**, 191–196.

8 R. D. Groot and T. J. Madden, *J. Chem. Phys.*, 1998, **108**, 8713–8724.

9 H. P. Erickson, *Biol. Proced. Online*, 2009, **11**, 32–51.

10 C. Cao, L. Zhang, B. Kent, S. Wong, C. J. Garvey and M. H. Stenzel, *Angew. Chem.*, 2021, **133**, 10430–10437.

11 B. Rupp, *Biomolecular Crystallography: Principles, Practice, and Application to Structural Biology*, Garland Science, 2009.

12 H. Liu, Y. L. Zhu, J. Zhang, Z. Y. Lu and Z. Y. Sun, *ACS Macro Lett.*, 2012, **1**, 1249–1253.

13 B. Y. Li, Y. C. Li and Z. Y. Lu, *J. Phys. Chem. Lett.*, 2020, **11**, 4542–4547.

14 H. M. Gao, H. Liu, H. J. Qian, G. S. Jiao and Z. Y. Lu, *Phys. Chem. Chem. Phys.*, 2018, **20**, 1381–1394.

15 J. Lu, Y. Xue, R. Shi, J. Kang, C. Y. Zhao, N. N. Zhang, C. Y. Wang and K. Lu, Z. Y.and Liu, *Chem. Sci.*, 2019, **10**, 2067–2074.

16 V. Gupta, S. Bhavanasi, M. Quadir, K. Singh, G. Ghosh, K. Vasamreddy, A. Ghosh, T. J. Siahaan, S. Banerjee and S. K. Banerjee, *J. Cell Commun. Signal.*, 2019, **13**, 319–330.

17 L. A. Frank, R. V. Contri, R. C. Beck, A. R. Pohlmann and S. S. Guterres, *Wiley Interdiscip. Rev. Nanomed. Nanobiotechnol.*, 2015, **7**, 623–639.

18 M. Li, N. T. Blum, J. Wu, J. Lin and P. Huang, *Adv. Mater.*, 2021, **33**, 2008438.

19 X. Q. Jia, L. Y. Wang and J. J. Du, *Nano Res.*, 2018, **11**, 5028–5048.

20 C. M. Hansen, *Hansen Solubility Parameters: A User's Handbook*, CRC press, 2007.

21 T. Lindvig, M. L. Michelsen and G. M. Kontogeorgis, *Fluid Ph. Equilibria*, 2002, **203**, 247–260.

22 R. D. Groot and T. J. Madden, *J. Chem. Phys.*, 1998, **108**, 8713–8724.

23 H. M. Gao, H. Liu, R. Zhang and Z. Y. Lu, *J. Phys. Chem. B*, 2019, **123**, 10311–10321.

24 H. M. Gao, L. Zhao, K. Liu and Z. Y. Lu, *J. Phys. Chem. Lett.*, 2021, **12**, 2340–2347.

25 Z. X. Yang, H. Q. Zhao, D. L. Wang, L. Yin, K. X. Cai, Z. H. Lin, T. Chen and C. F. Yang, *Phys. Chem. Chem. Phys.*, 2021, **23**, 19011–19021.

26 J. Zhang, Z. Y. Lu and Z. Y. Sun, *Soft Matter*, 2013, **9**, 1947–1954.

27 F. Wang, L. K. Feng, Y. D. Li and H. X. Guo, *Chin. J. Polym. Sci.*, 2023, **41**, 1392–1409.

28 T. Yamada, T. Yoshizaki and H. Yamakawa, *Macromolecules*, 1992, **25**, 377–383.

29 W. Lu, P. Yin, M. Osa, W. Wang, N. Kang, K. Hong and J. W. Mays, *J. Polym. Sci. B: Polym. Phys.*, 2017, **55**, 1526–1531.

30 R. Ramachandran, G. Beaucage, A. S. Kulkarni, D. McFaddin, J. Merrick-Mack and V. Galiatsatos, *Macromolecules*, 2008, **41**, 9802–9806.

31 H. Liu and Z. Y. Lu, *Front. Chem.*, 2011, **6**, 300–309.

32 H. Liu, H. Li and Z. Y. Lu, *Procedia Comput. Sci.*, 2011, **4**, 1021–1028.

33 H. Liu, Y. L. Zhu, Z. Y. Lu and F. Müller-Plathe, *J. Comput. Chem.*, 2016, **37**, 2634–2646.

34 T. Ouchi, T. Katsuura, M. Inaba, T. Azuma, Y. Hosaka and M. Imoto, *Polymer*, 1984, **25**, 412–416.

35 Y. V. Kissin, A. J. Brandolini and J. L. Garlick, *J. Polym. Sci., Part A:Polym. Chem.*, 2008, **46**, 5315–5329.

36 H. Arkın and W. Janke, *J. Chem. Phys.*, 2013, **138**, 054904.

37 V. Blavatska and W. Janke, *J. Chem. Phys.*, 2010, **133**, 184903.

38 V. Blavatska and W. Janke, *J. Chem. Phys.*, 2012, **136**, 104907.

39 M. Krone, S. Grottel and T. Ertl, 2011 IEEE Symposium on Biological Data Visualization (BioVis)., 2011, pp. 17–22.

40 R. X. Huang, R. P. Carney, K. Ikuma, F. Stellacci and B. L. Lau, *ACS Nano*, 2014, **8**, 5402–5412.

41 I. Sharma and S. K. Pattanayek, *Biophys. Chem.*, 2017, **226**, 14–22.

42 E. Lavagnini, J. L. Cook, P. B. Warren and C. A. Hunter, *J. Phys. Chem. B*, 2021, **125**, 3942–3952.

43 C. W. Tsai, C. I. Liu, Y. C. Chan, H. G. Tsai and R. C. Ruaan, *J. Phys. Chem. B*, 2010, **114**, 11620–11627.

44 A. S. Ausaf, H. M. Imtaiyaz, A. Islam and F. Ahmad, *Curr. Protein Pept. Sci.*, 2014, **15**, 456–476.

45 M. Z. Tien, A. G. Meyer, D. K. Sydykova, S. J. Spielman and C. O. Wilke, *PloS one*, 2013, **8**, e80635.

46 Y. Wang, J. Li, X. Li, B. Gao, J. Chen and Y. Song, *Sci. Rep.*, 2025, **15**, 8055.
